# Stereocontrolled 1,2-*cis* glycosylation as the driving force of progress in synthetic carbohydrate chemistry

**DOI:** 10.1039/c5sc00280j

**Published:** 2015-03-06

**Authors:** Swati S. Nigudkar, Alexei V. Demchenko

**Affiliations:** a Department of Chemistry and Biochemistry , University of Missouri – St. Louis , One University Blvd , St. Louis , MO 63121 , USA . Email: demchenkoa@umsl.edu

## Abstract

Recent developments in stereoselective 1,2-*cis* glycosylation that have emerged during the past decade are surveyed herein. For detailed coverage of the previous achievements in the field the reader is referred to our earlier reviews: A. V. Demchenko, *Curr. Org. Chem.*, 2003, **7**, 35–79 and *Synlett*, 2003, 1225–1240.

## Abstract 2

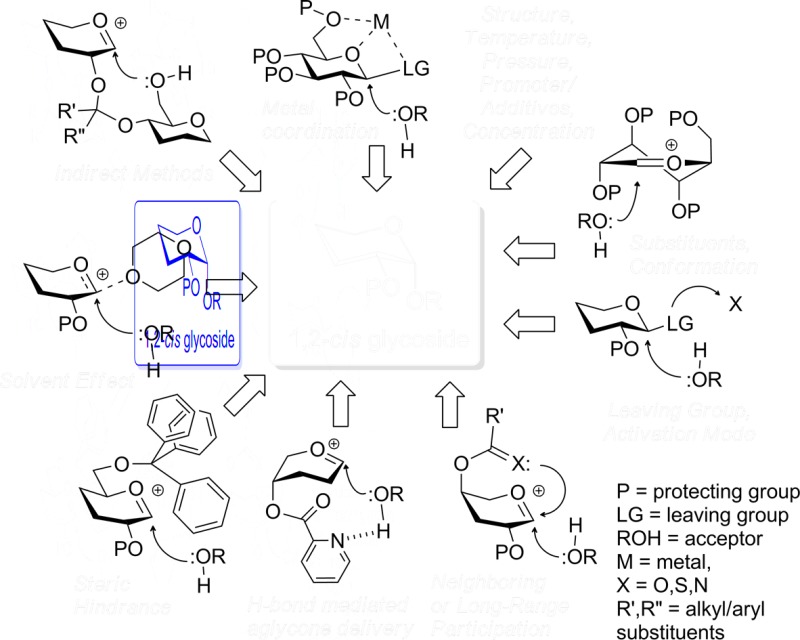
Recent developments in stereoselective 1,2-*cis* glycosylation that have emerged during the past decade are surveyed herein.

## A. Introduction

Carbohydrates, as polysaccharides or glycoconjugates, represent the largest class of naturally occurring compounds that are often found as essential components of many bioactive molecules in nature. Carbohydrates were initially viewed as energy-storage materials, structural components, and primary metabolites. Now it is known that carbohydrates mediate many fundamental biological processes such as immune defense, fertilization, metastasis, signal transduction, cell growth and cell–cell adhesion. In the past few years, we have been learning that carbohydrates play crucial roles in pathogenesis of diabetes, bacterial and viral infections, inflammation, development and growth of cancers, septicemia, and many other diseases. Clearly, uncovering the contributions of carbohydrates to cell biology would greatly facilitate advances in the field of glycosciences.^1^

For the most part, medicinally important carbohydrates exist as complex oligomers or as conjugates with other biomolecules including natural products, lipids, peptides, proteins, *etc.* ^2^ The carbohydrate part itself exists in various sizes and shapes ranging from monomeric sugars and simple linear chains to highly branched glycoforms. Major obstacles in studying natural carbohydrates are the difficulties in isolating, characterizing, and synthesizing these molecules due to their low abundance and heterogeneity in nature. While scientists have been able to successfully isolate and characterize certain classes of natural carbohydrates, the availability of pure isolates is still low. As a consequence, the systematic study of these molecules often relies on synthetic chemistry to provide pure compounds in significant quantities.

**Fig. 1:**
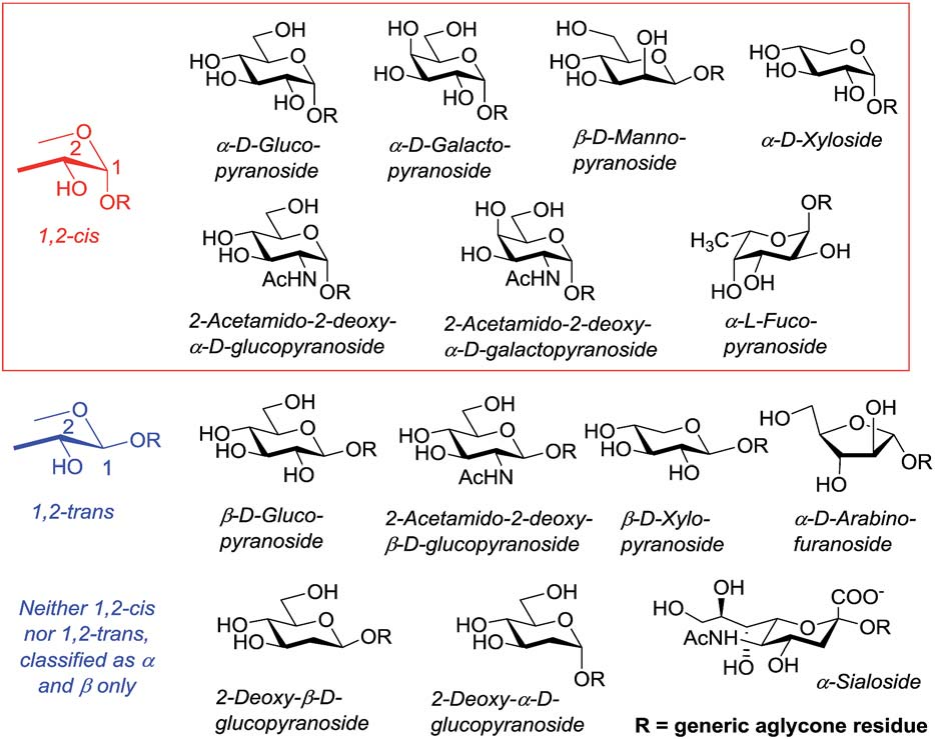
Common monosaccharide residues found in the mammalian and bacterial glycome.

Among the variety of glycosidic bonds in nature, it is the *O*-glycosidic bonds that are of major interest and challenge to chemists due to their high abundance and difficulty in synthesis. There are two major types of *O*-glycosides, which are, depending on nomenclature, most commonly defined as α- and β-, or 1,2-*cis* and 1,2-*trans* glycosides. Both 1,2-*cis* and 1,2-*trans* glycosides are important and abundant classes of linkages and are commonly found as components in a variety of natural compounds. However, it is 1,2-*cis* glycosyl residues, α-glycosides for d-glucose, d-galactose or β-glycosides for d-mannose, l-rhamnose, *etc.* that have proven to be synthetic hurdles for chemists. This review is dedicated to recent developments that have emerged to address the challenge of stereoselective 1,2-*cis* glycosylation. Some other common types of glycosides, for instance 2-deoxyglycosides and sialosides, lack the neighboring substituent. These compounds can neither be defined as 1,2-*cis* nor 1,2-*trans* glycosides, hence, these are commonly referred to as α- and β-glycosides. Representative examples of common glycosides are shown in Fig. 1.

**Fig. 2:**
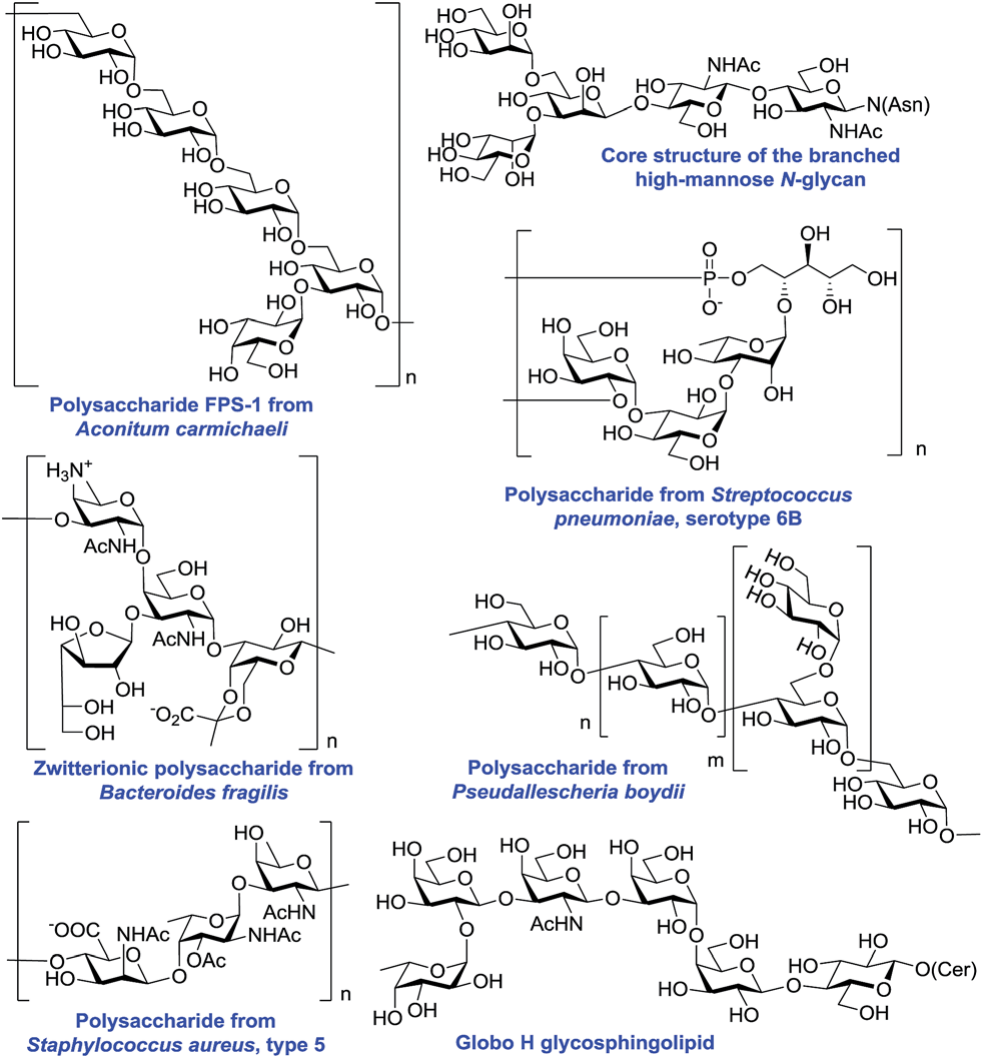
Naturally occurring oligosaccharides containing 1,2-*cis* linkages.

Many oligosaccharides containing 1,2-*cis O*-glycosidic linkages are of high importance due to their biological roles and therapeutic potential. Some representative naturally occurring oligosaccharides containing 1,2-*cis* linkages are shown in Fig. 2. For example, the immunomodulatory pentasaccharide FPS-1 from *Aconitum carmichaeli* is composed of an α-(1 → 6)-linked backbone with some α-(1 → 3) branching.^3^ The fungus *Pseudallescheria boydii* consists of a glycogen-like α-(1 → 4)-linked glucan backbone with occasional α-(1 → 6)-glucosyl branches.^4^ The zwitterionic polysaccharide A1 found on the capsule of the bacterium *Bacteroides fragilis* has a 1,2-*cis*-linked glycosaminoglycan motif.^5^ Many pneumococcal polysaccharides possess 1,2-*cis* glycosidic linkages, for instance a polysaccharide from *Streptococcus pneumonia* serotype 6B^6^ that is included in all current pneumococcal vaccines, has α-glucosyl and α-galactosyl residues. The trisaccharide repeating unit isolated from *Staphylococcus aureus* type 5^7^ possesses uncommon ManNAcA and FucNAc, both 1,2-*cis*-glycosidically linked. High mannose-type *N*-linked glycans^8^ that mediate the pathogenesis of many diseases bear an important 1,2-*cis*-linked β-mannosyl residue. All glycosphingolipids of the globoside family have an α-linked galactosyl residue and Globo-H, which is a current target for breast and prostate cancer vaccine development,^9^ has an α-fucosyl residue as well.

## B. Outline of chemical glycosylation: mechanism, general principles and special cases

**Scheme 1:**
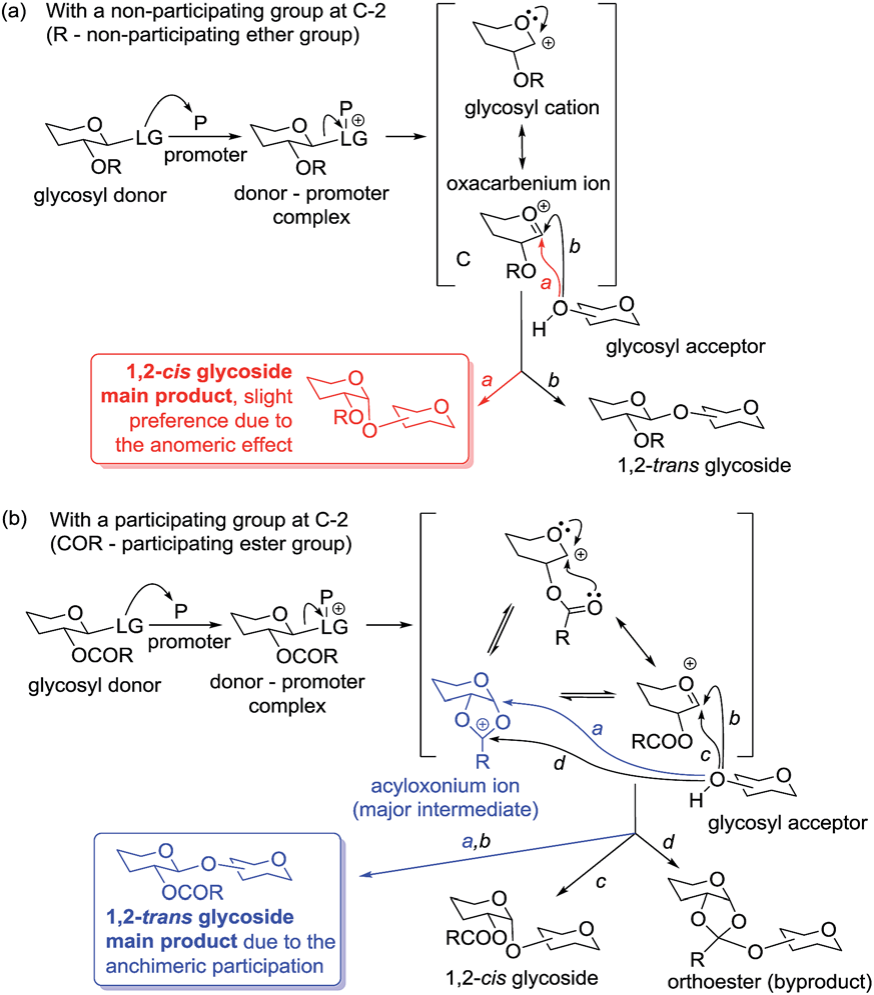
General outline of glycosylation and the key intermediates involved.

Glycosylation is arguably the most important, albeit challenging, reaction in the field of carbohydrate chemistry. Most commonly, it involves the reaction between a glycosyl donor and glycosyl acceptor, in the presence of an activator or promoter, to form a glycosidic bond. Upon activation, the promoter-assisted departure of the leaving group results in the formation of a glycosyl cation, which then gets stabilized *via* an oxacarbenium ion intermediate (Scheme 1a). The nucleophile, glycosyl acceptor, can then attack (to form the glycosidic bond) either from the top or the bottom face of the flattened ring. This would give rise to either 1,2-*trans* or 1,2-*cis* glycosides with respect to the neighboring substituent at C-2, and uncontrolled reactions may lead to a mixture thereof.

The formation of 1,2-*trans* linkages can be accomplished using the participatory effect of the neighboring 2-acyl substituent. In this case, the oxacarbenium ion can be further stabilized *via* a bicyclic acyloxonium intermediate, which becomes the key intermediate *en route* to glycosylation products (Scheme 1b). Since the bottom face of the ring is blocked, nucleophilic attack of the glycosyl acceptor would be directed from the opposite, top face. This typically provides access to the 1,2-*trans* linkage with very high or complete stereoselectivity. Occasionally, substantial amounts of 1,2-*cis*-linked products or orthoester formation are also observed.

While the stereoselective synthesis of 1,2-*trans* linkages can be reliably achieved with the use of neighboring group assistance,^10^ the formation of 1,2-*cis* linkages is typically much more challenging. The presence of a non-participating group is required for the synthesis of 1,2-*cis* glycosides, but the non-participating group alone cannot ensure the stereoselectivity. Although the α-product is favored by the anomeric effect,^11^ the stereoselectivity of glycosylation can be poor and requires other modes of stereocontrol. A variety of reaction conditions and structural elements of the reactants has been investigated. Although there are many examples wherein excellent 1,2-*cis* stereoselectivity of certain linkages has been achieved, no comprehensive method for 1,2-*cis* glycosylation is available.^12^

**Fig. 3:**
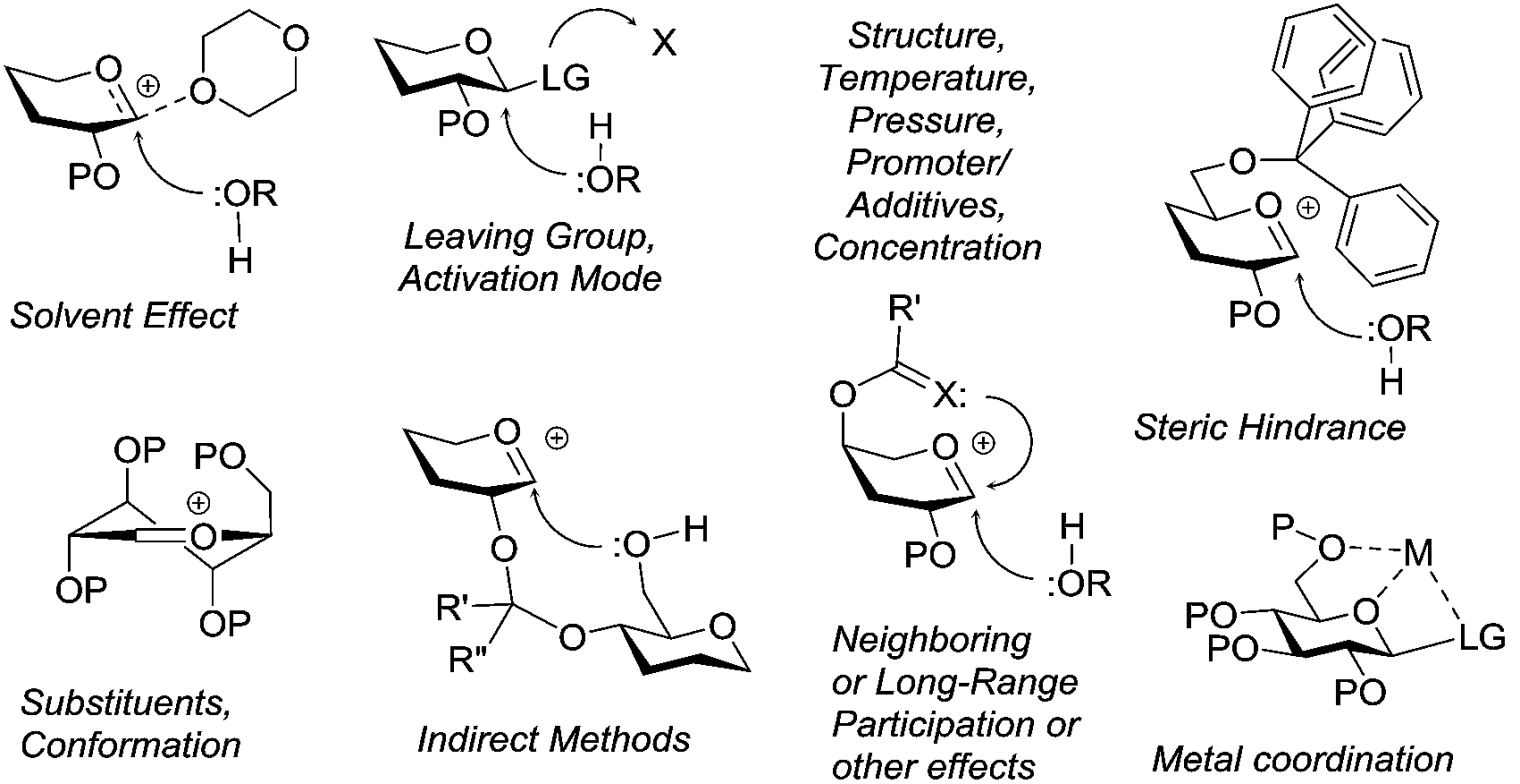
Factors affecting stereoselectivity.

In addition to the apparent complexity of the glycosylation process, there are other competing processes that cannot be disregarded. Side reactions, such as elimination, substitution (formation of unexpected substitution products or hydrolysis at the anomeric center), cyclization (inter and intramolecular orthoesterification), migration, redox, *etc.*,^13^ often complicate stereocontrol and compromise the yield of glycosylation. Several factors are known to affect the stereoselectivity and yield of glycosylation and those include temperature, solvent, type of donor used, type of acceptor used, amount and type of promoter used, protecting groups, *etc.* (Fig. 3). These effects and specifically designed methods to control the stereoselectivity of glycosylation will be discussed in the subsequent sections. While some sugars follow general trends, there are classes of compounds and glycosidic linkages that require special methods. These special cases of glycosylation require careful selection of techniques, their modification, or design of conceptually new approaches. Indirect or total synthesis-based technologies have been developed and applied specifically to the synthesis of these targets.

Glycosides of 2-amino-2-deoxy sugars, in particular those of the d-gluco and d-galacto series, are widely distributed in living organisms as glycoconjugates or glycosaminoglycans.^14^ Since a vast majority of naturally-occurring 2-amino-2-deoxy sugars are *N*-acetylated, from a synthetic point of view, a 2-acetamido-2-deoxy substituted glycosyl donor would be desirable. For this type of glycosyl donor, however, the oxacarbenium ion rearranges rapidly into an unreactive oxazoline intermediate. Therefore, even the synthesis of such 1,2-*trans* glycosides requires additional steps and a careful selection of suitable protecting groups. A minimal requirement for the synthesis of 1,2-*cis* glycosides would be the use of a C-2 non-participating moiety, most commonly azide. 2,3-Oxazolidinone protection introduced by Kerns and *N-p*-methoxybenzylidene protection explored by Nguyen also show good promise to become universal approaches to 1,2-*cis* glycosylation with 2-aminosugars (*vide infra*).

β-Mannosyl residues are frequently found in glycoproteins. The chemical synthesis of β-mannosides cannot be achieved by relying on the anomeric effect, which would favor axial α-mannosides. In addition, the formation of β-mannosides is further disfavored by the repulsive interactions that would have occurred between the axial C-2 substituent and the nucleophile approaching from the top face of the ring. For many years, the only direct procedure applicable to β-mannosylation – Ag–silicate promoted glycosidation of α-halides – was assumed to follow a bimolecular S_N_2 mechanism.^15^ The difficulty of direct β-mannosylation was addressed by developing a variety of indirect approaches such as C-2 oxidation–reduction, C-2 inversion, anomeric alkylation, and intramolecular aglycone delivery.^16^ This was the standing in the field before Crich and co-workers discovered that 4,6-*O*-benzylidene protected sulfoxide^17^ or thioglycoside^18^ glycosyl donors provide excellent β-manno stereoselectivity. Detailed mechanistic and spectroscopic studies by the Crich group^19^ showed that anomeric α-*O*-triflates generated *in situ* are reactive intermediates that can be converted into β-mannosides with high stereocontrol at low temperatures.

In comparison to their six-membered counterparts, furanosides are less abundant. Nevertheless, their presence in a variety of polysaccharides from plants, bacteria, parasites, and fungi makes this type of glycosidic linkage an important synthetic target.^20^ The synthesis of 1,2-*trans* furanosides is relatively straightforward and, similarly to that of pyranosides, can be reliably achieved with the use of glycosyl donors bearing a participating group at C-2. In contrast, the synthesis of 1,2-*cis* furanosides is difficult, even more so than with pyranosides due to the lack of anomeric effect and the conformational flexibility of the five-membered ring. In fact, both electronic and steric effects favor the formation of 1,2-*trans* furanosides. In the past decade, a notable improvement in 1,2-*cis* furanosylation was made possible with glycosyl donors in which the ring has been locked into a single conformation. These examples include 2,3-anhydro,^21^ 3,5-*O*-(di-*tert*-butylsilylene),^22^ and 3,5-*O*-tetraisopropyldisiloxanylidene^23^ protected bicyclic glycosyl donors. A recent example wherein stereoselective 1,2-*cis* glycofuranosylation was accomplished with the assistance of H-bond mediated aglycone delivery will be discussed below.

2-Deoxyglycosides are important constituents of many classes of antibiotics. The development of reliable methods for the stereoselective synthesis of both α- and β-2-deoxyglycosides is critical for the synthesis of natural products, drugs and glycomimetics.^24^ It should be noted that due to the lack of anchimeric assistance from the substituent at C-2, the synthesis of both types of linkages represents a notable challenge. Direct glycosylation of 2-deoxy glycosyl donors often results in the formation of anomeric mixtures, though notable recent progress in the area has to be acknowledged.^25,25g^ In spite of extensive efforts and notable progress, the chemical synthesis of sialosides also remains a significant challenge.^26^ The presence of a destabilizing electron-withdrawing carboxylic group and the lack of a participating auxiliary often drive sialylation reactions toward competitive elimination reactions resulting in the formation of a 2,3-dehydro derivative and/or in poor stereoselectivity (β-anomer). To overcome these problems, a variety of leaving groups and activation conditions for direct sialylation have been developed. It was also demonstrated that the *N*-substituent at C-5 plays an influential role in both the stereoselectivity of sialylation and the reactivity of sialyl donors.^26*d*^ A particular advance in recent years has been made with 4,5-*O*,*N*-oxazolidinone derivatives that provide high yields and stereoselectivities in sialylation.^27^

## C. Effect of the glycosyl donor

Glycosylations using trichloroacetimidates (TCAI)^28^ and thioglycosides^29^ as donors have become the most widely studied methods for chemical glycosylation. Our previous reviews on 1,2-*cis* glycosylation thoroughly discuss all pros and cons of using various leaving groups.^12a,12b^ Since glycosylation reactions commonly follow a unimolecular S_N_1 displacement mechanism, the orientation of the leaving group at the anomeric center is of little importance. However, occasionally glycosylation reactions proceed *via* an S_N_2-like mechanism with inversion of the anomeric configuration. The following leaving groups often provide excellent 1,2-*cis* selectivity: β-glycosyl halides formed from their α-counterparts with bromonium ions^30^ or from α-thioglycosides in the presence of bromine,^31^ glycosyl thiocyanates,^32^ and anomeric mannosyl triflates formed *in situ* from sulfoxides or thioglycosides for the synthesis of β-mannosides.^17,18^

It is well known that the stereoselectivity of glycosylation can be profoundly influenced by protecting groups.^33^ Neighboring protecting groups at C-2 traditionally known as participating groups for the synthesis of 1,2-*trans* glycosides can now assist in the formation of either 1,2-*cis* or 1,2-*trans* glycosides. Remote protecting groups at positions C-3, 4 and/or 6 may affect the stereoselectivity by means of participation, H-bond mediated aglycone delivery, steric hindrance and/or electron withdrawal. Also discussed in this section are protecting groups that restrict the conformational flexibility of carbohydrates or force carbohydrate molecules to adopt unusual conformations. Glycosidation of unprotected glycosyl donors with reactive glycosyl acceptors proceeding with good to excellent 1,2-*cis* stereoselectivity has also been reported.^34^

### C.1. Neighboring protecting group at C-2

**Scheme 2:**
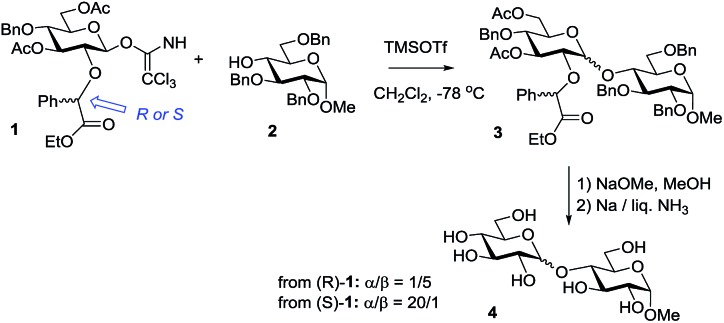
Stereoselective glycosylation with ethyl (*R*)- and (*S*)-mandelate protected glycosyl donor **1**.

As aforementioned, neighboring acyl-type protecting groups offer one of the most powerful tools to direct stereoselectivity toward the formation of a 1,2-*trans*-linked product. Demchenko and co-workers developed glycosyl donors equipped with a 2-picolinyl ether substituent that can also participate and form 1,2-*trans* glycosides stereoselectively.^35^ Boons and co-workers developed a participating group capable of participation from the opposite face of the ring giving rise to 1,2-*cis* linked glycosides.^36^ On activation of the glycosyl donor, the resulting oxacarbenium ion is attacked by a nucleophilic moiety *via* a six-membered intermediate. This attack, in principle, can lead to the formation of a *cis*- or *trans*-decalin-like system, and Boons and co-workers showed that the selectivity is highly dependent on the configuration of the asymmetric center of the chiral protecting group. To accommodate the bulky phenyl group in the pseudo-equatorial position of the newly formed six-membered ring, an auxiliary with (*S*)-stereochemistry would favor the *trans*-decalin-like intermediate. As a result, the nucleophilic attack of the glycosyl acceptor will occur from the bottom face leading to 1,2-*cis*-linked glycosides. Conversely, a chiral auxiliary with the opposite (*R*)-configuration could participate *via* the *cis*-decalin-like intermediate, thereby producing 1,2-*trans* glycosides. Ethyl mandelate was chosen to test this methodology because both the enantiomers are readily available, the conditions required for its installation are compatible with other protecting groups, and it is stable during glycosylation, but can be readily removed under mild reductive conditions. As depicted in Scheme 2, when an ethyl (*S*)-mandelate-protected donor (*S*)-**1** was glycosidated with glycosyl acceptor **2**, disaccharide **3** was obtained with high α-selectivity (α/β = 20/1). Conversely, when (*R*)-**1** was used as the glycosyl donor, a reversal of anomeric selectivity was observed (α/β = 1/5). Deprotection of the acyl groups using sodium methoxide in methanol and benzyl groups, including the chiral auxiliary, under Birch reduction conditions provided disaccharide **4**.

**Scheme 3:**
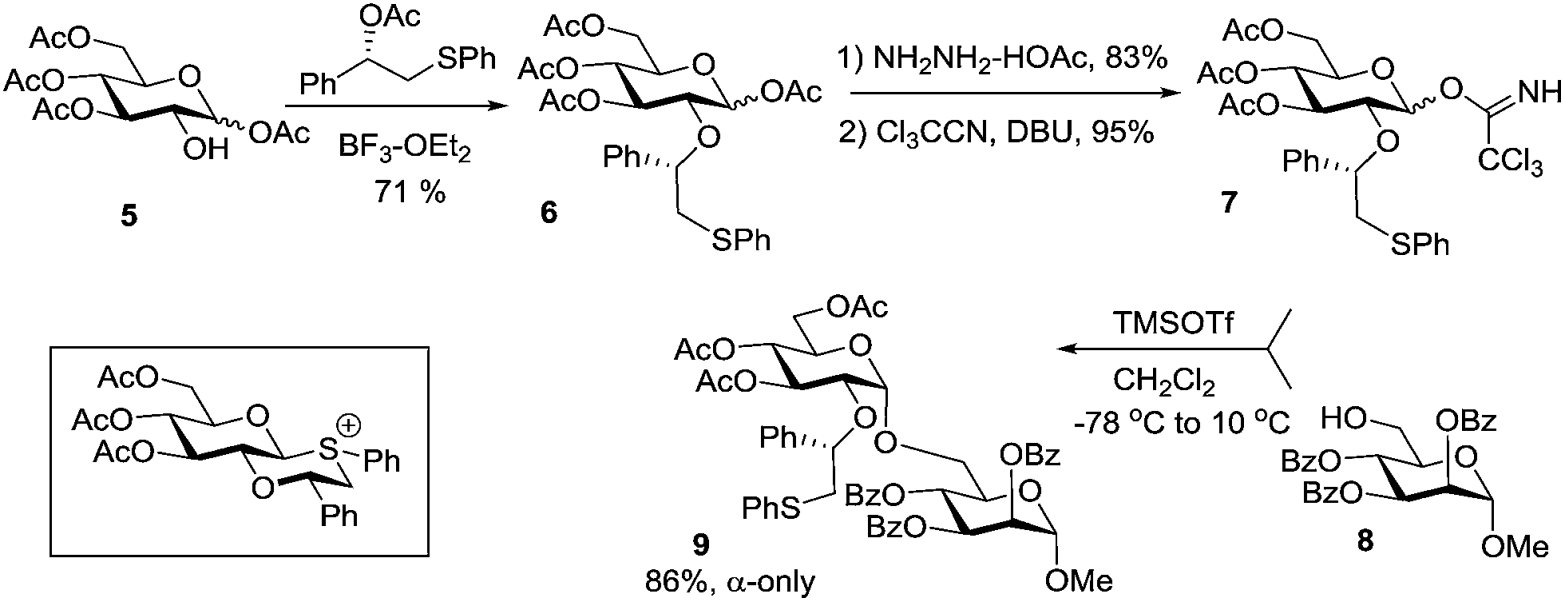
Synthesis of C-2 (*S*)-phenyl-thiomethylbenzyl ether-protected glycosyl donor **6** and its glycosidation.

The second generation auxiliary developed to further enhance 1,2-*cis* stereoselectivity was based on an (*S*)-phenyl-thiomethylbenzyl ether moiety at C-2 of the glycosyl donor.^37^ It was assumed that this type of moiety would be capable of more efficient and stereoselective participation *via* the formation of a chair and hence a more stable *trans*-decalin-like intermediate. In this case, the (*S*)-phenyl group will occupy the equatorial position to avoid unfavorable 1,3-diaxial interactions that would have occurred if the bulky phenyl group was placed in the axial position. As depicted in Scheme 3, 1-(*S*)-phenyl-2-(phenylsulfanyl)ethyl ether-protected TCAI donor **7** was obtained from glucose tetraacetate **5** *via* sequential protection, liberation of the anomeric hydroxyl and introduction of the imidoyl leaving group. Glycosyl donor **7** was then reacted with acceptor **8** in the presence of TMSOTf to afford α-glycoside **9** in 86% yield and with exclusive α-stereoselectivity. The auxiliary can then be removed by acetolysis in the presence of BF_3_–OEt_2_ and acetic anhydride. This method has been extended to the polymer-supported synthesis of the repeating unit of the immune-modulatory polysaccharide from *Aconitum carmichaeli* composed of an α-(1 → 6)-linked glucosyl backbone branched with α-(1 → 3)-linked glucosyl moieties.^38^

**Scheme 4:**
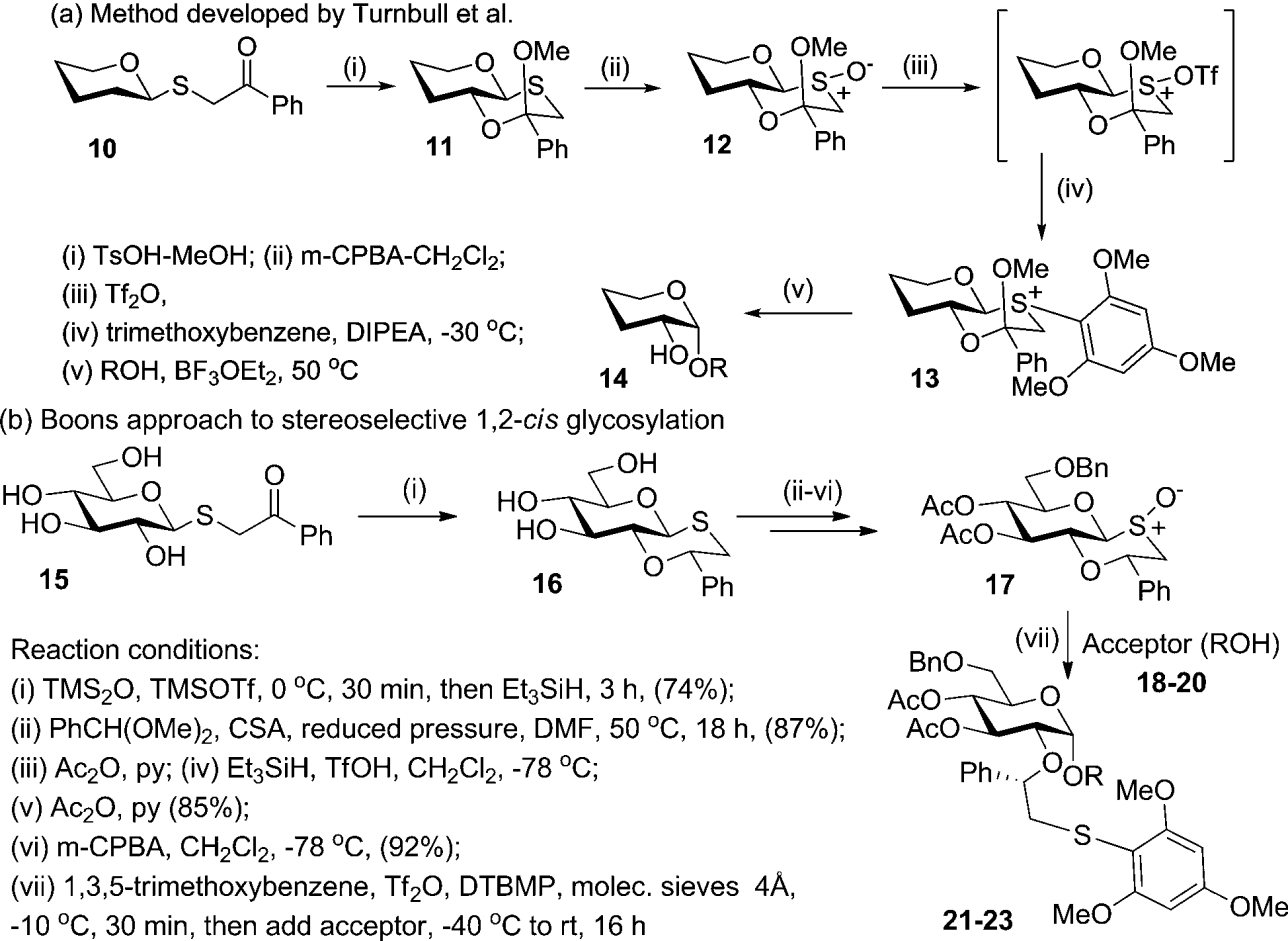
Stereoselective glycosylation *via* sulfonium ions.

More recently, to simplify this approach, Boons and co-workers adopted a different direction towards the synthesis of 1,2-*cis* glycosides.^39^ This was certainly inspired by their earlier work on chiral auxiliaries and inherent drawbacks related to the necessity of obtaining pure enantiomeric substrates. Additional inspiration came from work by Turnbull *et al.* who developed a very elegant approach using thioglycoside donors **10** having an anomeric α-directing group.^40^ As depicted in Scheme 4a, these reactions proceeded *via* bicyclic intermediate **11** that was activated *via* oxidation into sulfoxide **12** and *S*-arylation to form reactive sulfonium ion **13** *en route* to *O*-glycoside **14**.

In Boons’ approach depicted in Scheme 4b,^39^ sulfoxide donor **16** was prepared from thioglycoside **15** by treatment with trimethylsilyl anhydride (TMS_2_O) in the presence of TMSOTf, followed by reduction with Et_3_SiH. Compound **16** was then subjected to a series of protecting group manipulations followed by oxidation with *m*-CPBA to give sulfoxide **17**. Glycosidation of donor **17** included treatment with trifluoromethanesulfonic anhydride (Tf_2_O), arylation with 1,3,5-trimethoxybenzene, followed by the addition of glycosyl acceptors **18–20** to form the corresponding disaccharides **21–23** in high yields and stereoselectivities.^39^ It was observed that while the donors bearing electron-withdrawing groups at C-3, 4, and 6 gave only the α-anomer, their 4,6-diether substituted counterparts suffered from a slight loss of α-anomeric selectivity. This led to confusion that the highly reactive sulfonium ions partially react *via* the oxacarbenium ion intermediate.

Building upon their previous work, Turnbull and co-workers recently designed a new oxathiane donor scaffold where the axial methoxy group was replaced with an *O*-substituent constrained in a spirocyclic ring.^41^ As in the previous methods, the oxathiane spiroketal donor is then activated *via S*-arylation. Overall, the novel class of oxathiane glycosyl donors is easily accessible, highly α-selective in glycosylation, and offers high stability towards common protecting group manipulations.

### C.2. Remote protecting groups

The effects of remote substituents have long been considered of somewhat lesser importance than those of the neighboring substituent at C-2. However, the idea of participating groups at remote positions has been brought to attention by many researchers. There have been various reports, starting from long-range 6-*O*-acyl or carbonate group assisted synthesis of α-glucosides,^42^ both in favor and in opposition of the idea of remote participation. For derivatives of the d-galacto series a remote effect beneficial for the formation of α-galactosides was also noted when a participating moiety was present at C-4.^43^ Similar effects (including C-3 participation) were also detected for the derivatives of the l-fuco,^44^ l-rhamno,^45^ d-manno,^46^ and d-gluco^47^ series.

In 2009, Kim presented a dedicated study of the effect of 3- and 6-*O*-acetyl donors on the stereoselectivity of mannosylation.^48^ The comparative study indicated remote participation by 3-*O* and 6-*O* acetyl groups, but showed no participation by the 4-*O*-acyl group. Thus, when mannopyranosyl TCAI donors bearing electron-withdrawing ester groups, such as acetyl (**24**) or benzoyl (**25**) at the C-3 position, were coupled with primary acceptors **27–29** in the presence of TMSOTf, the corresponding disaccharides were obtained in excellent yields (88–94%) with high β-selectivity (α/β = 1/26–40, entries 1–4, Table 1). However, when benzyl sulfonyl was used as an electron-withdrawing group at C-3, the selectivity obtained with donor **26** was reversed and the corresponding disaccharides were obtained with preferential α-selectivity (α/β = 10–16/1, entries 5 and 6, Table 1).

**Table 1:** The effect of a 3-*O*-acyl protection on the stereoselectivity of mannosylation

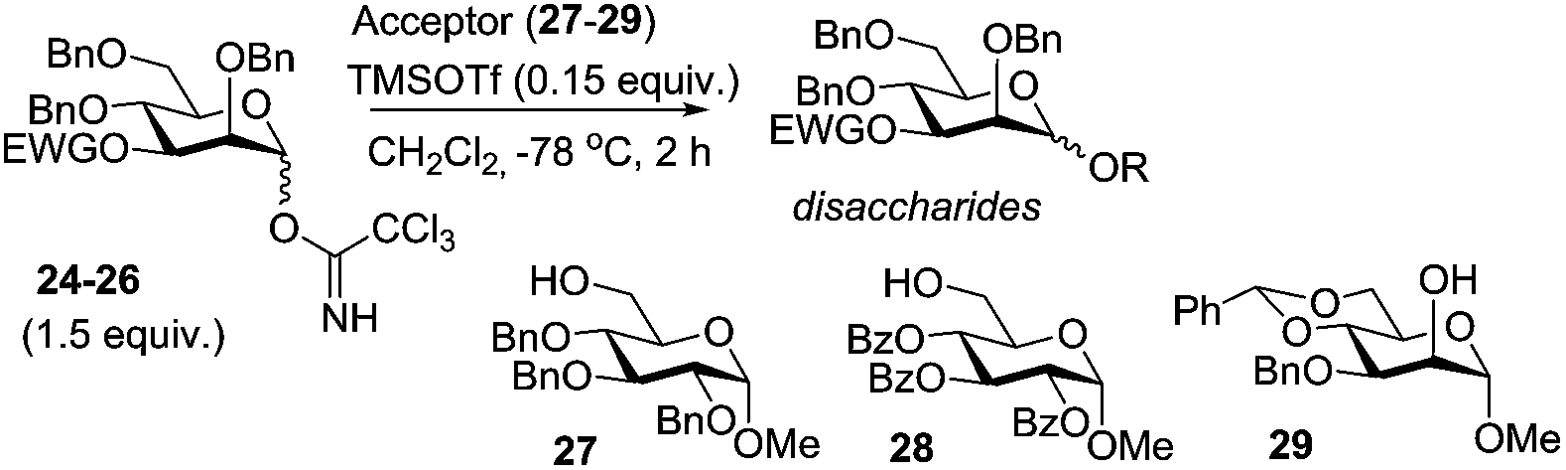				
Entry	Donor (EWG)	Acceptor (1.0 equiv.)	Disaccharide yield	α/β ratio
**1**	**24** (Ac)	**27**	91%	1/25.9
**2**	**24** (Ac)	**28**	94%	1/39.0
**3**	**24** (Ac)	**29**	92%	1/40.4
**4**	**25** (Bz)	**28**	88%	1/29.6
**5**	**26** (SO_2_Bn)	**27**	95%	15.9/1
**6**	**26** (SO_2_Bn)	**28**	93%	10.2/1

Very recently, Nifantiev *et al.* studied the effect of a 3-*O*-acyl substituent on the stereoselectivity obtained with either conformationally flexible or conformationally restricted glucosyl donors.^49^ As depicted in Table 2, when *N*-phenyltrifluoroacetimidate (PTFAI) donor **30** bearing acetyl groups at C-3 and C-6 was reacted with glycosyl acceptors **34** and **35** the corresponding disaccharides were obtained in good yields and with high selectivities (α/β = 5.3–11.2/1, entries 1 and 2). When glycosyl donor **31**, wherein the C-6 acetyl was replaced with C-6 benzoyl, was used, a further increase in selectivity was observed (α/β = 16.4/1, entry 3). In this context, 3,6-di-*O*-acetyl protected sulfoxide donor **32** provided lower yield and stereoselectivity (entry 4). A similar selectivity, albeit excellent yield, was observed with conformationally restricted 4,6-*O*-benzylidene-protected glucosyl donor **33** (96% yield, α/β = 5.9/1, entry 5). The effect of steric bulkiness or strong electron-withdrawing properties of remote substituents, particularly those at C-6, have been known for a while. The beneficial effect of such substituents on 1,2-*cis* glucosylation and galactosylation was attributed to shielding (steric or electronic) of the top face of the ring, therefore favoring nucleophilic attack from the opposite side.^15b,50^

**Table 2:** The effect of a 3-*O*- and 6-*O*-acyl protection on the stereoselectivity of glucosylation^*a*^

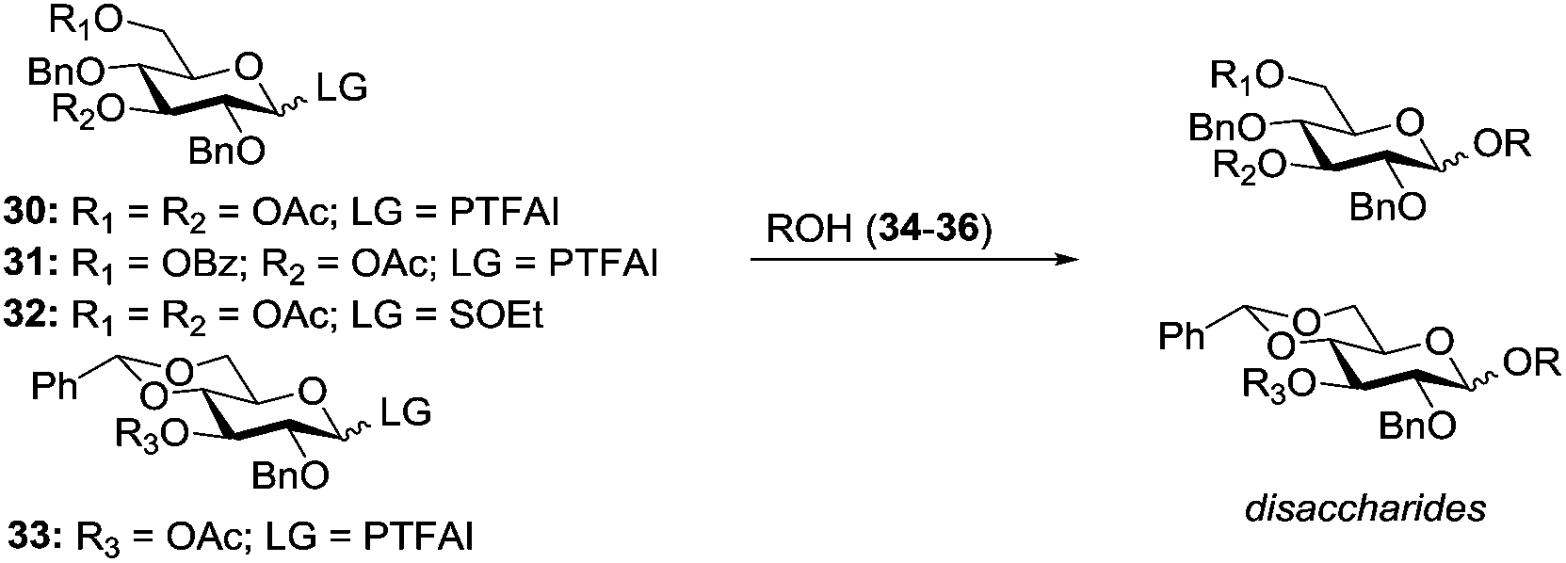			
Entry	Donor	Acceptor	Yield, α/β ratio
1^a^	**30**	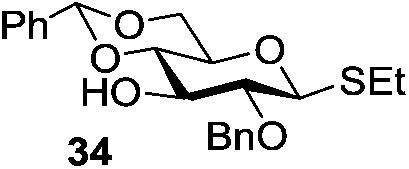	89%, 5.3/1
2^a^	**30**	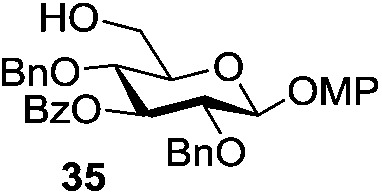	93%, 11.2/1
3^a^	**31**	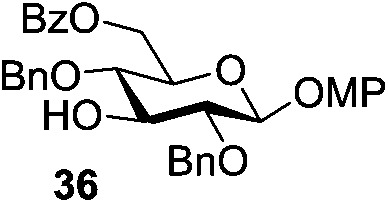	93%, 16.4/1
4^b^	**32**	**34**	59%, 6.8/1
5^c^	**33**	**35**	96%, 5.9/1

^*a*^Conditions: ^a^MeOTf, CH_2_Cl_2_, AW-300, –35 → –15 °C; ^b^Tf_2_O, DTBMP, CH_2_Cl_2_, –78 → 0 °C; ^c^MeOTf, CH_2_Cl_2_, AW-300, 20 °C.

A recent study with 2-azido-2-deoxy sugars revealed an interesting relationship between the stereoselectivity and the effect of remote participating groups in GalN_3_ and GlcN_3_ sugars.^51^ Over the course of this study it was observed that for GlcN_3_ sugars, acetyl groups at C-3 and C-6 positions show more α-directing effects whereas 4-*O*-acetyl is more β-directing.^52^ Crich showed that bulky 3-*O-tert*-butyldimethylsilyl (TBDMS) can push the axial 2-*O*-benzyl of mannosyl donors towards the anomeric center, thereby hindering nucleophilic attack from the top face, leading to poor β-selectivity.^53^ On the other hand, naphthylpropargyl ether protection at C-2 or C-3 favors high β-manno selectivity.^54^ Hung and co-workers developed a series of orthogonally protected d-glucoaminyl donors for stereoselective introduction of α-linkages into heparin-related sequences.^55^ The most advantageous protecting group pattern was determined to be a 2-azido functionality, 2-naphthylmethyl (2-NAP) group at C-4, and *p*-bromobenzyl (*p*-BrBn) at C-3, and TBDPS at C-6 positions. The α-directing effect of 4-*O-p*-BrBn and 6-*O*-TBDPS groups was deemed to be steric, preventing the attack of a glycosyl acceptor from the unwanted top face.

**Scheme 5:**
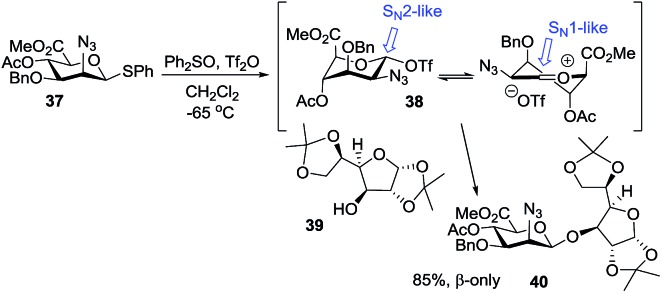
Rationalization of the high β-selectivity achieved with 2-azidomannouronate donor **37**.

Codee and co-workers investigated the use of a 2-azidomannouronate ester donor for glycosidation, and observed high 1,2-*cis* selectivity.^56^ On gaining an insight into the reaction mechanism, it was concluded that when thiophenyl donor **37** is activated in the presence of diphenyl sulfoxide and triflic anhydride, anomeric triflate **38** is formed (Scheme 5). The latter exists as an interchangeable mixture of conformers with the ^1^C_4_ chair as the predominant species. In principle, triflate **38** can lead to the β-linked product *via* an S_N_2-like displacement. Alternatively, the reaction can proceed *via* an S_N_1-like pathway. In this case, the oxacarbenium ion intermediate will preferentially adopt the ^3^H_4_ half-chair conformation, which closely resembles the major ^1^C_4_ conformation of triflate **38**. In this case, the C-5 carboxylate occupies a pseudo-axial position allowing for stabilization of the positive charge. The incoming nucleophile **39** will then attack from the β-face to produce disaccharide **40** with complete 1,2-*cis* selectivity in 85% yield.

A very different stereodirecting effect was discovered for remote picolinyl (Pic) and picoloyl (Pico) substituents. As aforementioned, a picolinyl at C-2 formally participates at the anomeric center and gives 1,2-*trans* glycosides *via* the six-membered ring intermediate.^35*b*^ The action of the remote picolinyl and related picoloyl substituents is totally different. Not being able to participate at the anomeric center directly, picolinyl nitrogen forms a hydrogen bond with the incoming glycosyl acceptor. As a result, a very high facial selectivity, always *syn* in respect to the picolinyl substituent, is observed.^57^ This rather unexpected involvement of remote picolinyl substituents was termed as H-bond-mediated aglycone delivery (HAD). Based on the above hypothesis, it was shown that under high dilution conditions (5 mM), 4-*O*-picoloyl or picolinyl glucosyl donors (**41–45**) provide faster reaction times and enhanced selectivity compared to those obtained in standard concentration (50 mM). Thus, glucosyl donors **41** and **42** provided high levels of α-selectivity, particularly with *O*-picoloyl protection (α/β = >25/1, entry 1, Table 3). Galactosyl donor **43** and rhamnosyl donor **44** gave high β-selectivity (α/β = >1/25, entries 3 and 4, respectively). As an extension to this study, Demchenko and co-workers showed that the presence of a 3-*O*-picoloyl group in mannosyl donor **45** can effectively provide β-mannosides with high stereoselectivity at room temperature (α/β = 1/18.5, entry 5).^58^

**Table 3:** H-bond-mediated aglycone delivery (HAD)

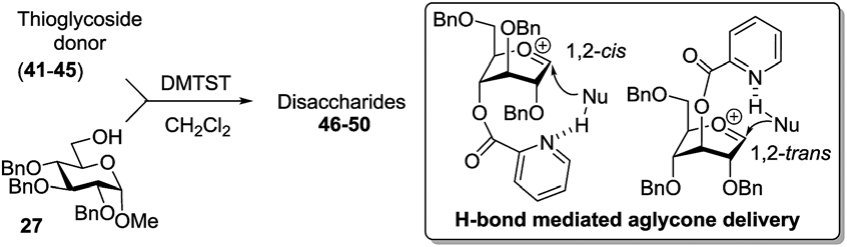					
Entry	Donor	Conc. **27**	Time	Product (yield)	α/β ratio
1	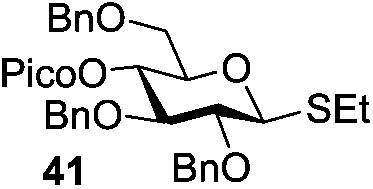	5 mM	4 h	**46** (73%)	>25/1
2	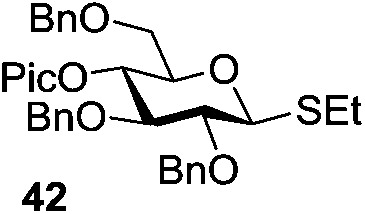	5 mM	5 h	**47** (86%)	5.3/1
3	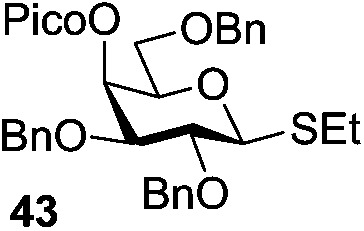	5 mM	1 h	**48** (95%)	>1/25
4	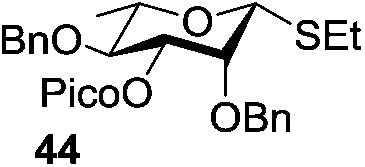	50 mM	15 min	**49** (94%)	>1/25
5	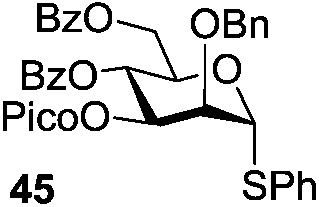	5 mM	2.5 h	**50** (91%)	1/18.5

**Scheme 6:**
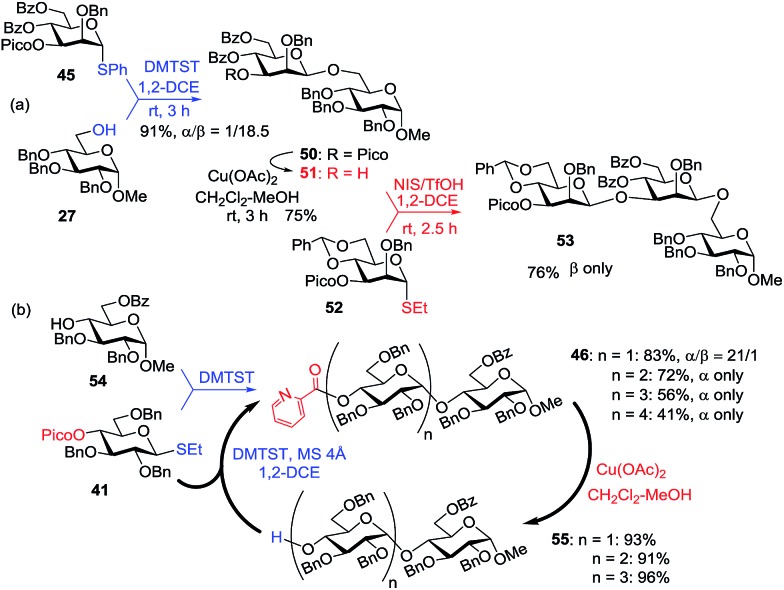
HAD synthesis of β-mannan and α-glucan.

The applicability of this approach was demonstrated for the synthesis of oligosaccharide **53** containing both primary and secondary β-mannosidic linkages (Scheme 6a). Thus, when 3-*O*-picolylated mannosyl donor **45** was reacted with glycosyl acceptor **27** in the presence of DMTST, β-linked disaccharide **50** was obtained with α/β = 1/18.5 selectivity. The 3-*O*-picoloyl group of **50** was then selectively removed using copper(ii) acetate and the resulting acceptor **51** was coupled with mannosyl donor **52**, to provide the desired trisaccharide **53** in 76% yield and with complete β-selectivity.

Further application of the HAD method has resulted in the synthesis of linear and branched α-glucans.^59^ As depicted in Scheme 6b, when 4-*O*-picoloyl glucosyl donor **41** was glycosylated with acceptor **54** in the presence of DMTST, disaccharide **46** was obtained in 83% yield (α/β = 21/1). The 4-*O*-picoloyl group was then selectively removed with copper(ii) acetate to form the second generation glycosyl acceptor **55**. The process was repeated to obtain pentasaccharide **46** (*n* = 4) with 41% yield and complete α-selectivity.

At first, the HAD approach was limited to *S*-ethyl glycosyl donors and only in the presence of DMTST, in high dilution, and low temperature. Other leaving groups gave much lower stereoselectivity.^60^ Combining the mechanistic studies of the HAD reaction and bromine-promoted glycosylations (*vide infra*) Yasomanee and Demchenko devised a very effective method that allows for highly stereoselective α-glucosidation of practically all common leaving groups (*S*-phenyl, *S*-tolyl, *S*/*O*-imidates) at regular concentrations and ambient temperature.^60^ Young and co-workers extended the HAD approach to β-stereoselective d- and l-arabinofuranosylation.^61^ In this case, 5-*O*-(2-quinolinecarbonyl) substituted arabinose was employed as the glycosyl donor. Mong and co-workers successfully applied 6-*O*-picoloylated glycosyl donors to the synthesis of β-2-deoxy glycosides.^25*g*^

### C.3. Conformation-restraining cyclic protecting groups

Torsional effects induced by cyclic protecting groups may also strongly affect the stereoselectivity of glycosylation. The best-known example of this effect is the work by Crich and co-workers on the synthesis of β-mannosides.^62^ Thus, it has been demonstrated that 4,6-*O*-benzylidene-protected thioglycoside donors give superior β-manno selectivity in comparison to that achieved with donors lacking this type of protection.^63^ The stereoselectivity observed was rationalized by carrying out experiments in which the benzylidene protected sulfoxide donor^64^ is pre-activated using Tf_2_O to form a sulfonium salt, which collapses into the α-triflate that exists in dynamic equilibrium with the contact ion pair. The presence of glycosyl triflate intermediates in mannosylation was also recognized with thioglycoside,^65^ TCAI,^66^ 2-(hydroxycarbonyl)-benzyl,^67^ hemiacetal,^68^ pentenoate,^69^ and phthalate^70^ donors, all protected as 4,6-benzylidene acetals. It is believed that the closely associated triflate counterion shields the α-face and β-linked product forms preferentially. An α-deuterium kinetic isotope effect (KIE) study indicated substantial oxacarbenium ion character of this reaction pathway, ruling out the possibility of a bimolecular displacement.^62*b*^ Similar conclusions were made as a result of KIE experiments with mannosyl iodides.^71^ The deactivating effect of benzylidene substituents was found to be a combination of torsional strain,^72^ restricting the conformational flexibility of the ring, and enhanced electron-withdrawal.^73^ The latter effect is due to locking the hydroxymethyl group in the conformation wherein the C6–O6 bond is directed away from *O*-5. This may cause additional destabilization of the oxacarbenium intermediate that seeks for compensation from tight coordination to the counter anion.

While the study of 4,6-*O*-benzylidene protected glycopyranosyl triflates revealed high β-selectivities with mannosyl donors, high α-selectivity is obtained with glucosyl donors (Table 4).^62*a*^ This finding was rationalized by the fact that the α-triflate intermediate undergoes equilibrium with its more reactive β-counterpart rather than with the oxacarbenium ion intermediate. The rate and equilibrium constant for the formation of β-glucosyl triflate are such that it preferentially forms the α-linked product.

**Table 4:** Stereodirecting effect of 4,6-*O*-benzylidene acetal

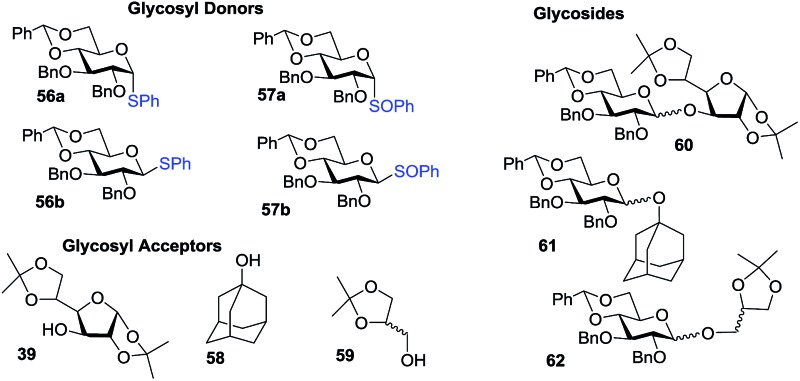				
Entry	Donor, acceptor	Coupling reagent	Product	Yield, α/β ratio
1	**56a**, **39**	PhSOTf	**60**	70%, >95/5
2	**56b**, **39**	PhSOTf	**60**	80%, >95/5
3	**57b**, **58**	Tf_2_O	**61**	63%, >95/5
4	**57a**, **59**	Tf_2_O	**62**	89%, >95/5

**Scheme 7:**
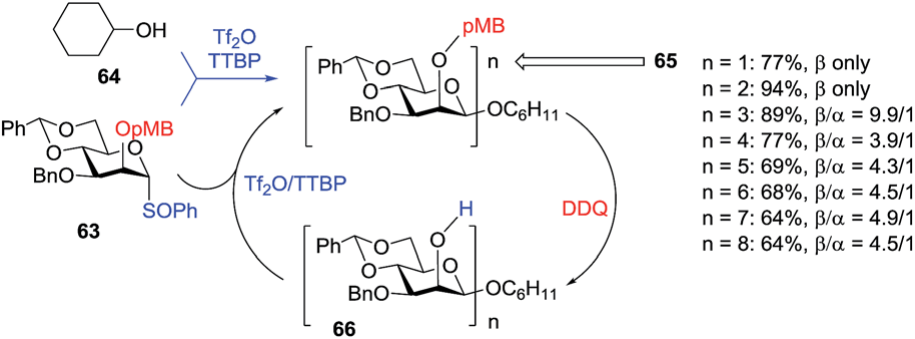
β-Linked mannans by the sulfoxide protocol.

Many useful applications have evolved from the Crich methodology for β-mannosylation. For instance, the direct syntheses of β-(1 → 2)- and β-(1 → 4)-mannans represent the power of this technique.^74^ As depicted in Scheme 7, synthesis of the (1 → 2)-mannan was achieved by means of the sulfoxide coupling protocol. Thus, 2-*O*-paramethoxybenzyl protected sulfoxide donor **63** was reacted with cyclohexanol **64** in the presence of triflic anhydride and 2,4,6-tri-*tert*-butylpyrimidine (TTBP) to afford β-mannoside **65** (*n* = 1) in 77% yield. The latter was deprotected with DDQ to give glycosyl acceptor **66**. Repetition of glycosylation-deprotection steps led to a series of (1 → 2)-linked homologs. For instance, octasaccharide **65** (*n* = 8) was obtained in 64% yield (β/α = 4.5/1). In this context, the (1 → 4)-linked mannan was prepared from thioglycoside donors activated using sulfinamide methodology.

To study the influence of similar conformationally rigid protecting groups, on the selectivity obtained, Werz and co-workers synthesized a variety of mannosyl donors with a spiroannulated cyclopropane ring at C-5 bearing one hydroxyl group.^75^ It was shown that the cyclopropane group leads to fixation of the chair-like conformation, similar to that shown for 4,6-benzylidene protected sugars although high β-selectivity was not achieved.

**Scheme 8:**
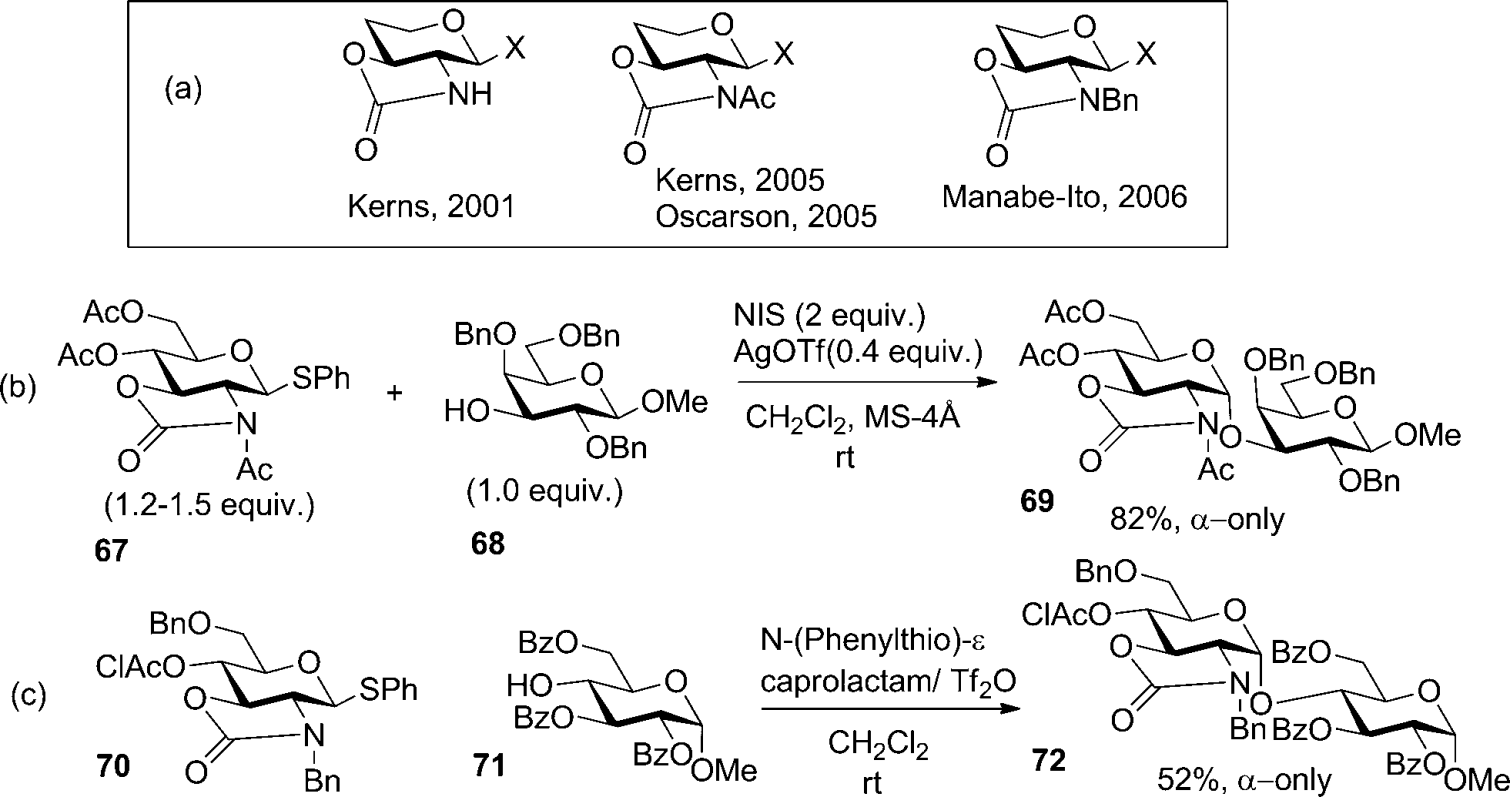
Selective α-glycosylation with *N*-acetyl- and *N*-benzyl-2,3-oxazolidinone-protected donors **67** and **70**.

Kerns discovered that 2,3-*trans*-oxazolidinone-protected glucosaminyl donors provide excellent 1,2-*cis* selectivity in glycosylations (Scheme 8a).^76^ Although high α-selectivity could be obtained, the oxazolidinone protected donor showed propensity to undergo side reactions, such as *N*-glycosylation or *N*-sulfenylation. To rectify this, Kerns *et al.* ^77^ and Oscarson *et al.* ^78^ reported the use of *N*-acetylated oxazolidinones.^76a,77a^ These donors showed switchable stereoselectivity in glycosylation that was achieved by tuning the reaction conditions.^79^ This interesting finding stimulated further studies. Mechanistically it was suggested that the β-linked product is formed initially, which rapidly anomerizes into the corresponding α-anomer. The presence of the oxazolidinone ring is the key for this anomerization to occur, which was found to proceed *via* endocyclic C1–O5 bond cleavage.^80^ For instance, when *N*-acetyl-2,3-oxazolidinone protected donor **67** was reacted with glycosyl acceptor **68** in the presence of NIS and AgOTf, disaccharide **69** was obtained in 82% yield (α-only, Scheme 8b). Manabe, Ito and their co-workers reported *N*-benzylated 2,3-oxazolidinone donors for 1,2-*cis* glycosylation.^81^ Thus, when glycosyl donor **70** was glycosidated with acceptor **71** in the presence of *N*-(phenylthio)-ε-caprolactam and triflic anhydride, disaccharide **72** was obtained in 52% yield with complete α-selectivity (Scheme 8c).

Crich *et al.* showed that the 2,3-*O*-carbonate protecting group is highly α-selective for mannosylation and rhamnosylation.^53,82^ In contrast, 3,4-*O*-carbonate protected rhamnosyl donors showed moderate β-selectivities owing to the electron withdrawing but non-participating nature of this group. Crich also reported the synthesis of β-glucosides using 2,3-*O*-carbonate protected glucosyl donors.^83^ It was suggested that the conformation restricting *trans*-fused ring favors the formation of an α-triflate intermediate over the formation of an oxacarbenium ion. The effect of 3,4-*O*-carbonate protection was found to be weaker with a slight preference toward β-selectivity.^25*d*^ Ye and co-workers studied 2,3-*O*-carbonyl protected glucose and galactose donors for pre-activation-based glycosylation.^84^ These reactions were generally β-stereoselective, but Lewis acid additives were found to favor α-stereoselectivity (*vide infra*). A beneficial effect of a bulky 4,6-*O*-di-*tert*-butylsilylene (DTBS) protecting group^85^ on α-selective galactosylation and galactosamination was recently applied to the synthesis of a series of human ABO histo-blood group type 2 antigens by Kiso and co-workers.^86^

## D. Effect of the glycosyl acceptor

Many examples wherein different glycosyl acceptors have different selectivities can be seen throughout the text of this review. A rule of thumb is that the alcohol reactivity is inversely correlated with the stereoselectivity and the most reactive hydroxyls give the lowest α/β-ratios: the stronger the nucleophile, the faster the reaction, and therefore the more difficult it is to control its outcome. As an example, glycosylation of the axial 4-OH of galactose often gives excellent 1,2-*cis* stereoselectivity. Occasionally, primary hydroxyls provide higher stereoselectivity in comparison to that of secondary hydroxyl groups. This can serve as evidence for the glycosylation reaction proceeding *via* a bimolecular mechanism, at least partially. Primary alcohols also gave higher stereoselectivity in H-bond-mediated aglycone delivery reactions mediated by remote picolinyl groups.^57^

**Scheme 9:**
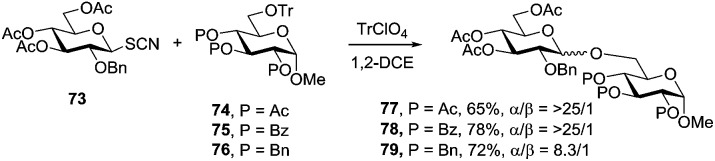
Acyl groups in acceptors enhance stereoselectivity.

It is well-established that ester electron-withdrawing substituents reduce the electron density of the neighboring hydroxyl group, lowering its nucleophilicity.^87^ This may improve stereoselectivity, as the reaction can be carried out in a more controlled manner. Recently, Demchenko and co-workers have shown that electron-withdrawing acyl protecting groups have a dramatic effect on the stereoselectivity obtained with thiocyanates as glycosyl donors.^88^ Thus, when thiocyanate **73** was reacted with acyl-protected acceptors **74** and **75**, the corresponding disaccharides **77** and **78** were obtained with complete α-selectivity (α/β = >25/1, Scheme 9). However, when benzyl-protected acceptor **76** was used instead, the stereoselectivity dropped (**79**, α/β = 8.3/1).

Very recently, Toshima and co-workers reported a novel approach that makes use of the chiral recognition of aglycones.^89^ Thus, in glycosylations of racemic alcohols in the presence of a chiral Brønsted acid activator, one enantiomer was glycosylated preferentially and the glycosides were obtained with high stereoselectivity and yields.

## E. Effect of the reaction conditions

### E.1. Temperature

Kinetically controlled glycosylations at lower temperatures generally favor β-glycoside formation,^90^ although converse observations have also been reported.^91^ Since the α-glycoside is thermodynamically favored due to the anomeric effect, it is predominantly formed at high temperatures. A number of examples have been presented throughout other parts of this review.

### E.2. Solvent

**Scheme 10:**
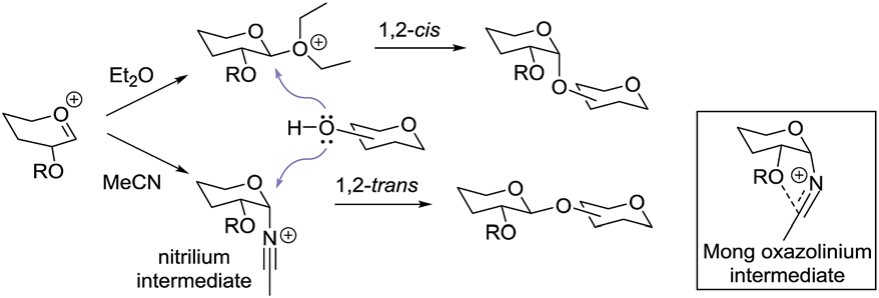
Effect of the reaction solvent.

The effect of reaction solvent on the selectivity of the glycosylation reaction has been widely studied. In general, polar reaction solvents increase the rate of β-glycoside formation *via* charge separation between *O*-5 and β-*O*-1. If the synthesis of α-glycosides is desired, CH_2_Cl_2_, ClCH_2_CH_2_Cl or toluene would be suitable candidates as the reaction solvent. However, there are more powerful forces than simple solvation that have to be taken into consideration. It has been shown that ethereal solvents have a tendency to drive glycosylation in an α-selective fashion, while nitrile solvents increase the amount of β-glycoside formation.^42b,92^ These observations were rationalized as follows: ether type reaction solvents such as diethyl ether,^93^ tetrahydrofuran,^93^ or dioxane^94^ lead to the preferential formation of the equatorial intermediate. On the other hand, if the reactions are performed in acetonitrile, the nitrilium cation formed *in situ* exclusively adopts an axial orientation, allowing stereoselective formation of equatorially substituted glycosides (Scheme 10). This approach permits the formation of 1,2-*trans* glucosides with good stereoselectivity even with glycosyl donors bearing a non-participating substituent.

Recently, the Mong group proposed a revised mechanism for glycosylation in nitrile solvents.^95^ Accordingly, the oxacarbenium ion intermediate interacts with the nitrile solvent producing mixtures of α- and β-glycosyl nitrilium intermediates. Though the formation of 1,2-*cis* nitrilium species is favored by the anomeric effect, it is further reinforced through the participation of *O*-2 (Scheme 10). The resulting glycosyl oxazolinium intermediate is then attacked by a nucleophile from the top face leading to formation of the β-product.

Many applications of solvent systems controlling reaction stereoselectivity are known. A representative example shown in Table 5 makes use of an *N*-trichloroacetyl carbamate leaving group introduced by Redlich^96^ and Vankar.^97^ Omura *et al.* showed that the stereoselectivity of glycosylation can be reversed by simply switching the solvent.^98^ Thus, when *N*-trichloroacetyl carbamate **81** was glycosidated with acceptor **28** in the presence of TMSClO_4_ in diethyl ether as the solvent, disaccharide **82** was formed with high α-selectivity (entry 1). Conversely, high β-selectivity could be achieved by activation with TMSOTf in EtCN (entry 2). Apparently, this example makes use of the promoter and temperature effects.

**Table 5:** One-pot synthesis and glycosidation of carbamates

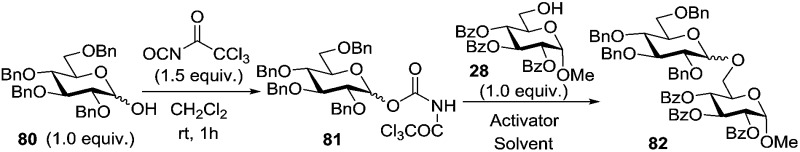				
Entry	Activator	Solvent	Reaction conditions	Yield, α/β ratio
1	TMSClO_4_ (1.5 equiv.)	Et_2_O	0 °C, 0.5 h	99%, 93/7
2	TMSOTf (1.5 equiv.)	EtCN	–40 °C, 0.5 h then –23 °C, 0.5 h	88%, 8/92

**Scheme 11:**
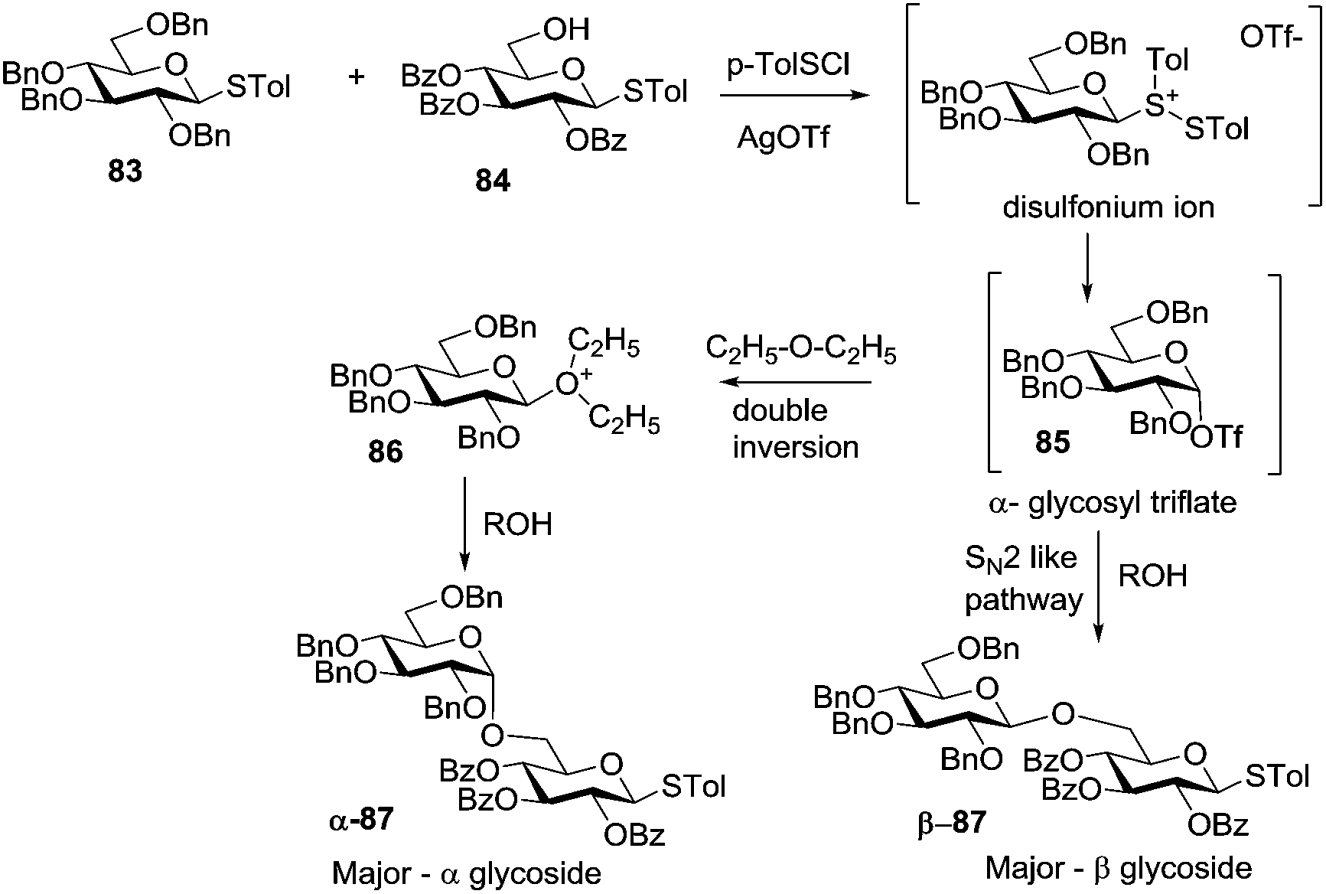
The solvent effect on preactivation-based glycosylation.

Huang *et al.* have recently studied the solvent and additive effects on the stereochemical outcome of the thioglycoside-based glycosylation strategy.^99^ When donor **83** was pre-activated with *p*-TolSOTf, formed *in situ* from *p*-TolSCl and AgOTf (3 equiv.) in diethyl ether disaccharide **87** was obtained in 67% yield (α/β = 1.1/1, Scheme 11). When the amount of AgOTf was decreased to 1.1 equiv., significant change in α-selectivity was observed (α/β = 6/1). In addition, when the reaction was performed by increasing the volume of diethyl ether 10 fold, further enhancement in α-selectivity was observed (α/β = 10/1). On the other hand, when dichloromethane was used as the reaction solvent, the stereoselectivity was switched (α/β = 1/8). With the belief that glycosyl triflates are formed as the key reaction intermediates, the observed stereoselectivity was rationalized as follows. The reactions performed in diethyl ether proceed through a double-inversion mechanism. Under dilute conditions and with lower excess of AgOTf, solvent participation becomes more effective, resulting in higher α-selectivity. In the case of dichloromethane, due to the non-nucleophilic nature of the solvent, the reaction is likely to proceed *via* an S_N_2-like triflate displacement pathway leading to β-glycosides (Scheme 11).

Ito and co-workers developed a high-throughput screening system to study the synergistic solvent effect of combined ethereal and halogenated solvents on the course of glycosylation.^92^ This study employed the use of glycosyl donors, which were isotopically labeled with per-deuterated protecting groups: benzyl ether (Bn-*d*_7_) and *d*_10_-cyclohexylidene ketal. The labeled donor was glycosidated in the presence of MeOTf as the activator and 2,6-di-*tert*-butyl-4-methylpyridine (DTBMP) in various solvents.

**Scheme 12:**
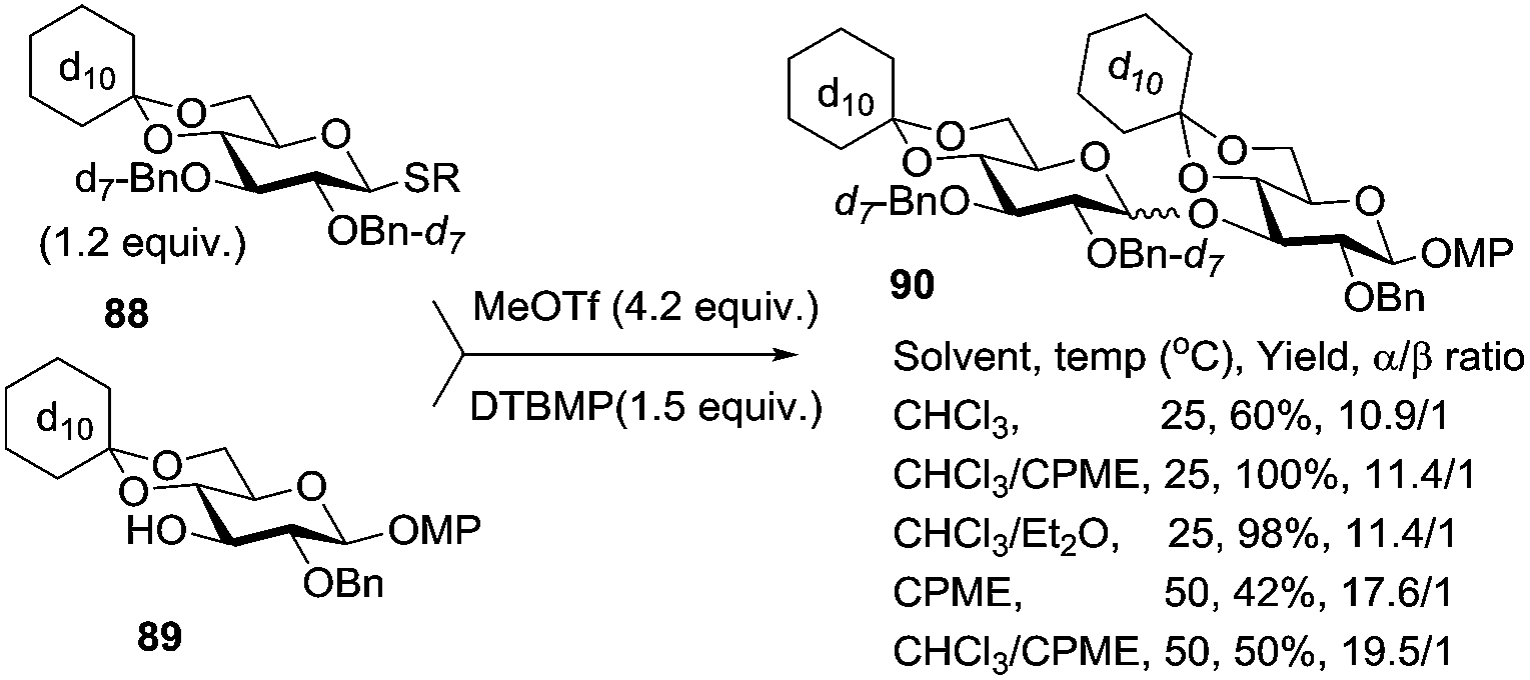
Solvent and temperature effects.

As depicted in Scheme 12, when per-deuterated benzyl ether protected thioglycoside donor **88** was reacted with per-deuterated glycosyl acceptor **89** in the presence of methyl triflate (MeOTf) as a promoter, disaccharide **90** was obtained with selectivity up to α/β = 19.5/1. A mixture of CHCl_3_/Et_2_O or CHCl_3_/cyclopentyl methyl ether (CPME) 1/1 (v/v) provided the best results and the use of such solvent systems was extended to the synthesis of a variety of 1,2-*cis* linkages.^92,100^ The beneficial effect of high temperature on α-selectivity has also been noted. The advantage of using Bn-*d*_7_ is the “disappearance” of all benzylic methylene signals at around 4–5 ppm, thereby making it easier to interpret the proton NMR spectra of the products.

Mong and co-workers took a different direction in studying the reaction solvent effect by using dimethylformamide (DMF) as a co-solvent, a rather uncommon reaction solvent in glycosylations.^101^ This study employed two conceptually different protocols for glycosylation. First, a conventional method (procedure A, Table 6), wherein a mixture of glycosyl donor, acceptor, and DMF was activated with NIS and TMSOTf. As shown in Table 6, reaction of benzylated donor **91** with acceptor **92** gave moderate stereoselectivity (82%, α/β = 6/1, entry 1) in the presence of 1.5 equiv. of DMF. The increase in the amount of DMF to 3 and 6 equiv. (entries 2 and 3, respectively) translated into a significant increase in α-stereoselectivity (up to α/β = 19/1, entry 3).

**Table 6:** Investigation of DMF-mediated glycosylations

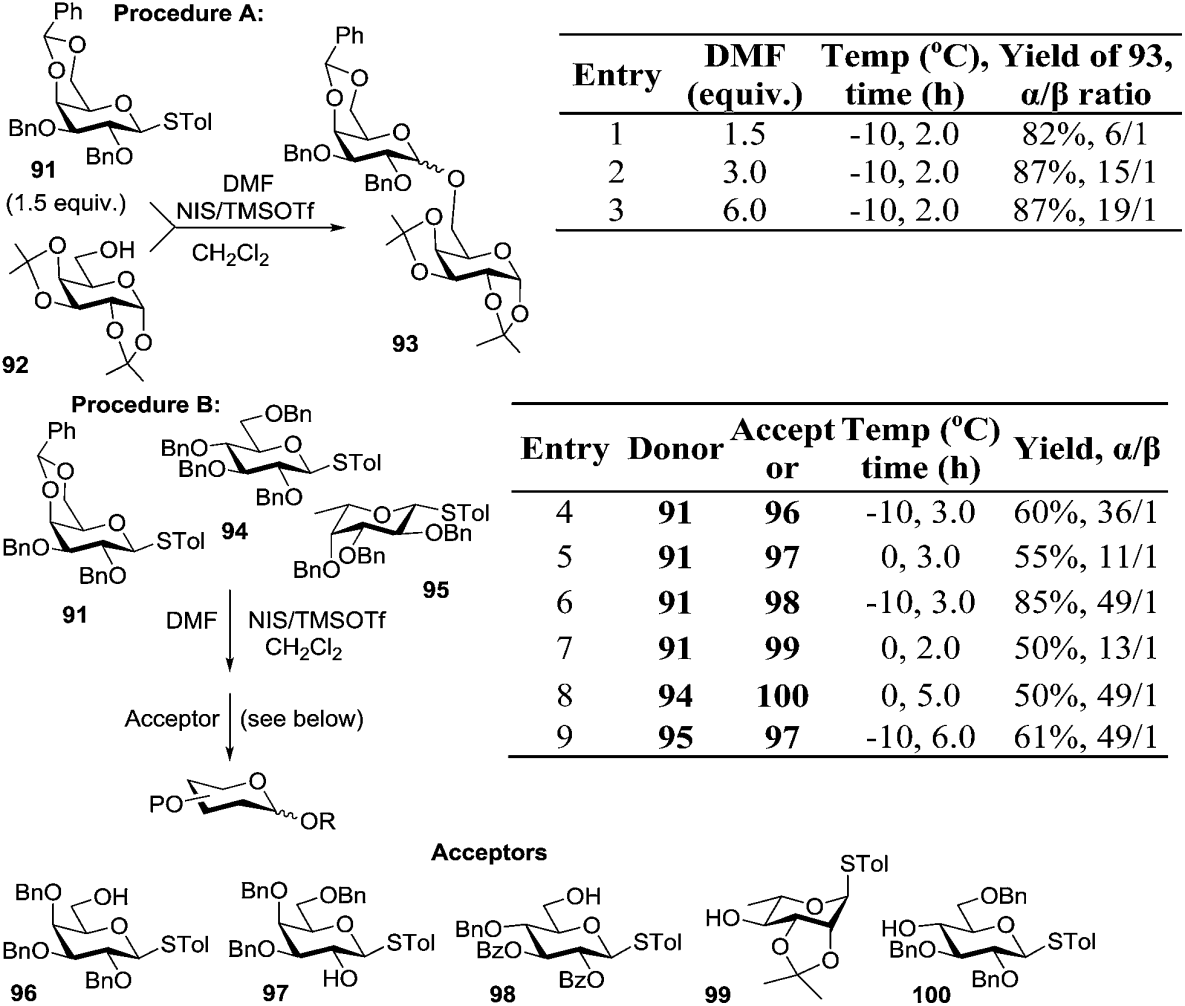

The results obtained using procedure **A** were then applied to the investigation of the effectiveness of the pre-activation based glycosylation procedure **B**. Accordingly, the glycosyl donor was reacted with NIS and TMSOTf in the presence of DMF followed by the addition of the glycosyl acceptor. All glycosylations between thioglycoside donors **91**, **94**, or **95** and acceptors **96–100** proceeded with very high α-selectivity (α/β = 11–49/1, entries 4–9, Table 6).

**Scheme 13:**
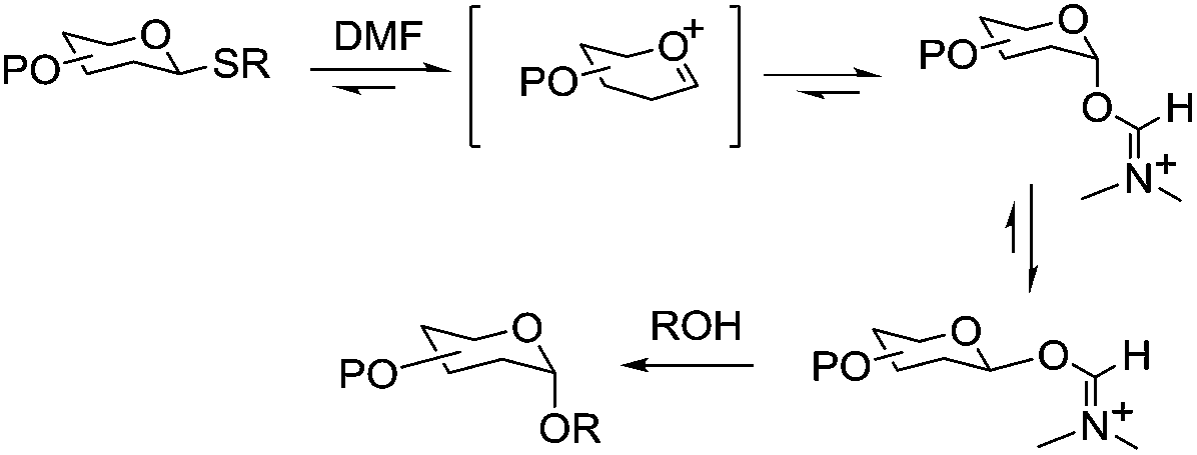
DMF-mediated glycosylation.

This modulating effect of DMF, which was particularly evident in the preactivation-based protocol (procedure **B**) was rationalized as follows. DMF involvement traps the glycosyl oxacarbenium ion resulting in an equilibrating mixture of α/β-glycosyl *O*-imidates (Scheme 13). The more reactive β-imidate will react faster with the glycosyl acceptor producing the desired α-glycoside with high selectivity. This procedure implies an S_N_2-like inversion *en route* to the products of glycosylation. Interestingly, the use of ethereal solvents had no effect on the further improvement of stereoselectivity, irrespective of the type of ethereal solvent used.

**Scheme 14:**
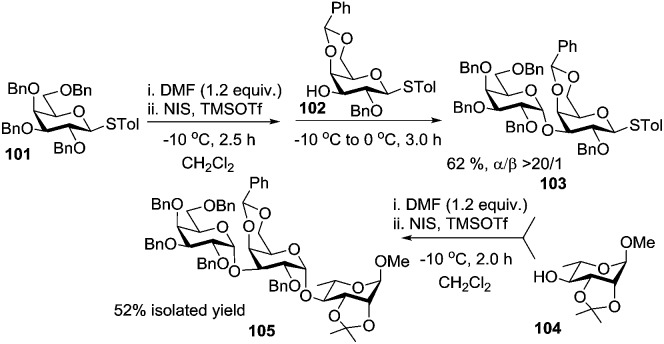
DMF-mediated synthesis of trisaccharide **105**.

Encouraged by the α-stereodirecting effect of DMF, the preactivation protocol was then extended to a sequential one-pot oligosaccharide synthesis.^101^ As depicted in Scheme 14, trisaccharide **105** containing two contiguous 1,2-*cis* linkages was efficiently assembled in an overall yield of 52% from building blocks **101**, **102** and **104**. An interesting feature of DMF as an additive to the one-pot multi-step synthesis is that it is regenerated after the first coupling and hence can be engaged in the subsequent modulation cycles.

### E.3. Promoter, additives, and chelators

Many decades ago, glycosylation of poorly nucleophilic acceptors was sluggish and inefficient.^102^ Early attempts to improve the glycosylation process by Zemplen^103^ and Helferich^104^ also revealed the necessity to find a delicate balance between the reactivity and stereoselectivity because it was noted that faster reactions often result in decreased stereoselectivity and *vice versa*.^105^ It has become general knowledge that milder activating conditions are beneficial for 1,2-*cis* glycosylation. Thus, halide ion-catalyzed reactions gave the best results for glycosylation with glycosyl bromides^30^ and iodides.^106,107^

**Scheme 15:**
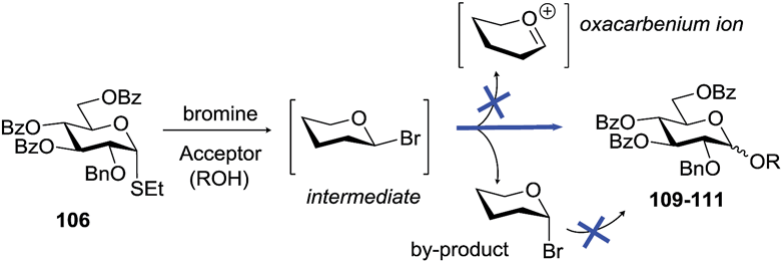
Stereoselective glycosidation of superdisarmed thioglycoside **106** *via* reactive β-bromide intermediate.

Thioglycosides often give higher selectivity when activated with a mild promoter, such as iodonium dicollidine perchlorate (IDCP).^108^ Recently, Demchenko and co-workers investigated the glycosidation of thioglycosides in the presence of bromine, another mild activator.^31^ It was demonstrated that bromine-mediated glycosylation of thioglycoside **106** leads to exclusive α-selectivity in products **109–111** (entries 1a, 2a and 3a, Scheme 15). This reaction was monitored by NMR, showing that β-bromide is the reactive intermediate which, however, can undergo a rapid anomerization into the α-linked counterpart. Once formed, the α-bromide is totally unreactive under the established reaction conditions, so the yield of glycosylation can be low with secondary alcohols (entries 2a and 3a). It was also shown that the α-bromide can be reactivated in the presence of a mercury(ii) additive. This pathway was found to be very beneficial for the glycosylation of secondary alcohols (entries 2b and 3b), but can compromise the α-selectivity of glycosylation with primary alcohols (entry 1b).

While many of the current methodologies for glycosylation require the use of stoichiometric amounts of promoters, the use of transition metal catalysts helps to achieve greener glycosylation and offers new opportunities for stereocontrol.^109^ Nguyen and co-workers studied palladium(ii)-catalyzed glycosidation of TCAI donors using Pd(CH_3_CN)_4_(BF_4_)_2_ or similar catalysts.^110^ This study evolved into the investigation of a series of nickel catalysts providing an efficient means for the glycosidation of *N-p*-methoxybenzylidene-protected 2-amino-2-deoxy TCAI donor.^111^ The nature of the ligand on nickel has been found to be the deciding factor in controlling the stereoselectivity of glycosylation. Thus, it was observed that electron-withdrawing substituents help to decrease the reaction time, which is translated into increased α-selectivity. The efficiency of nickel-catalyzed reactions was extended to the synthesis of a number of challenging targets. As summarized in Table 7, *N*-benzylidene TCAI donor **112** bearing different *para* substituents was reacted with primary (**27–28**) and secondary glycosyl acceptors (**58**, **113–114**) under catalysis of Ni(4-F-PhCN)_4_(OTf)_2_, to provide disaccharides (**115–119**) with very high α-selectivity.

**Table 7:** Nickel-catalyzed glycosidation of donor **112**

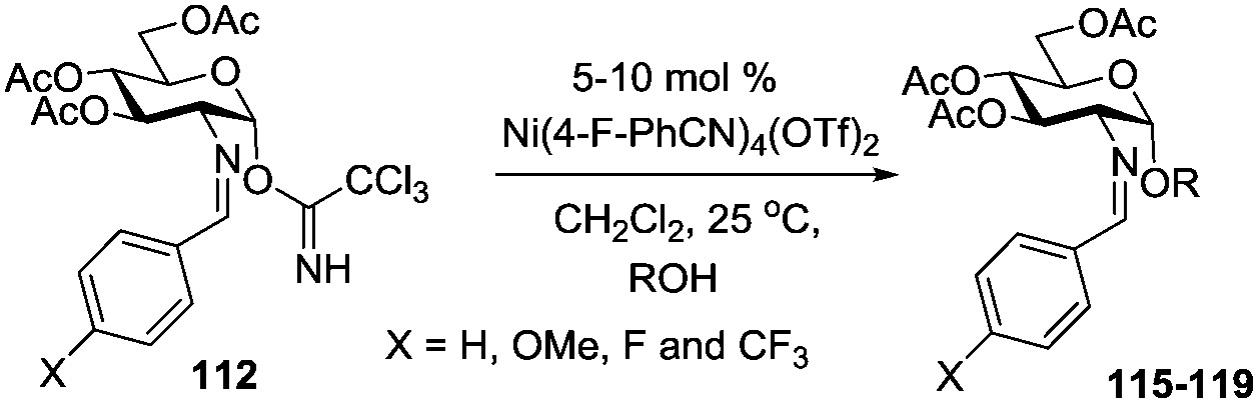		
Entry	R–OH	Product, yield, α/β ratio
1	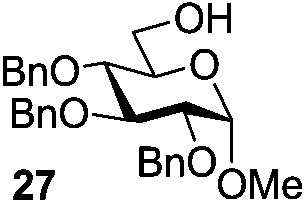	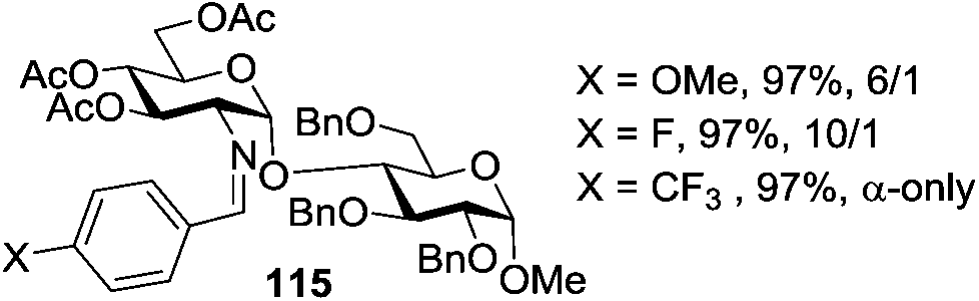
2	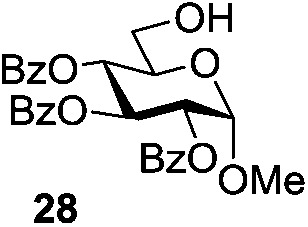	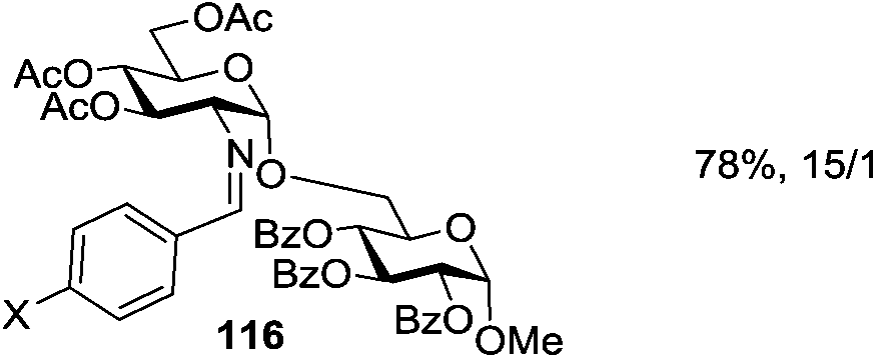
3	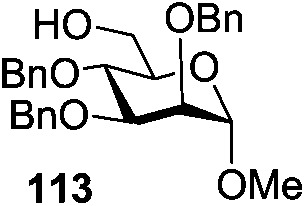	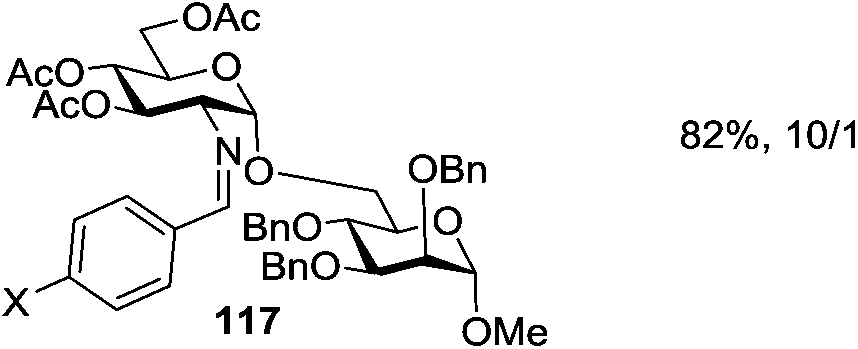
4	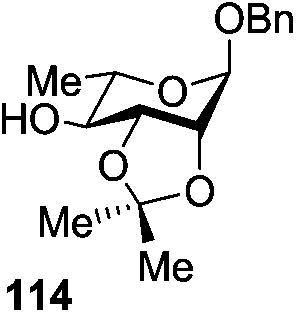	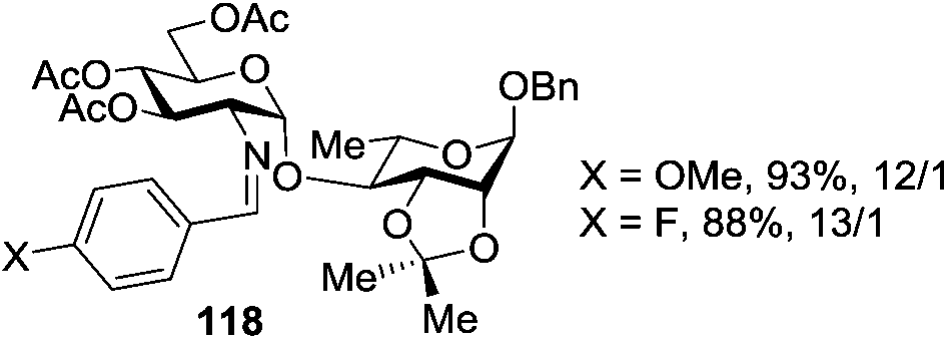
5	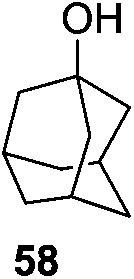	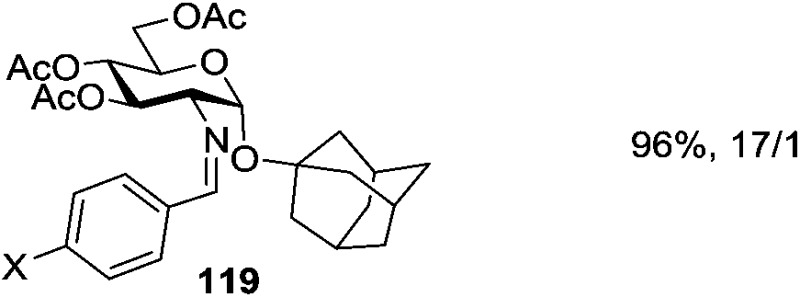

Recently there has been an explosion in the study of gold-catalyzed activation of alkynes to exploit the low oxophilic character of gold and the excellent functional group compatibilities these catalysts exhibit.^112^ This includes work by Hotha and co-workers where propargyl glycosides were activated using Au(iii) chloride to give α/β mixtures of glycosides and disaccharides in good yields. Yu and co-workers conducted a similar study with glycosyl *ortho*-alkynylbenzoates under catalytic Au(i) activation conditions.^112d,112e^ Another promising new field is the use of chiral thioureas as organocatalysts for glycosylation.^113^ As of now, this approach is limited to the synthesis of 2-deoxy α-glycosides^114^ and β-selective glycosylation with 2-oxygenated sugars.^115^

**Scheme 16:**
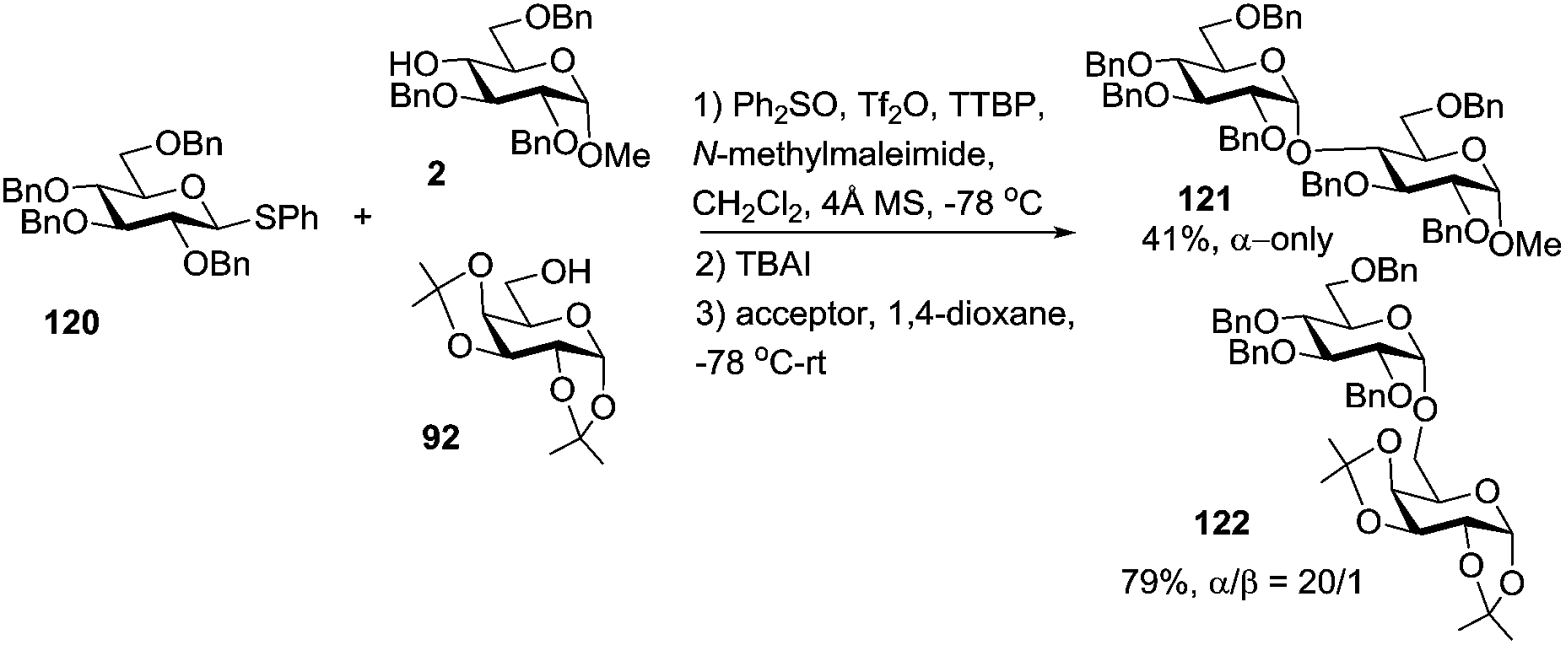
Synthesis of 1,2-*cis*-linked glycosides by activation of thioglycosides in the presence of TBAI.

Bennett and co-workers recently investigated the activation of thioglycosides with Ph_2_SO in the presence of TBAI. It was observed that this reaction proceeds *via* the intermediacy of glycosyl iodides.^116^ The underpinning idea of using TBAI is that the conversion of α-glycosyl triflates into β-glycosyl iodides would favor the formation of α-glycosides. Thus, when *S*-phenyl donor **120** was preactivated using Ph_2_SO/Tf_2_O followed by the addition of TBAI and glycosyl acceptors **2** or **92**, the respective disaccharides **121** (41%) or **122** (79%) were obtained in excellent or even complete α-stereoselectivity (Scheme 16).

Various additives to promoter systems often influence the stereochemical outcome of glycosylation. Amongst the most remarkable examples is the use of perchlorate ion additive that was found to be very influential in 1,2-*cis* glycosylation.^117^ Very recently, the effectiveness of the use of silver perchlorate as the activator in glycosidation of thioimidates and thioglycosides to provide better 1,2-*cis* selectivity than that achieved with more common triflates, has been studied.^118^ While studying 2,3-*O*-carbonyl protected glucose and galactose donors, which are generally β-stereoselective, Ye and co-workers observed that Lewis acid additives favor α-stereoselectivity in preactivation-based glycosylation.^84^ Thus, a catalytic amount of BF_3_–OEt_2_ or AgBF_4_ as well as 1 equiv. of AgPF_6_ or SnCl_4_ completely reversed the stereoselectivity to give α-linked products. It was assumed that similar to that proven for 2,3-oxazolidinones,^80^ the initially formed β-linked product anomerizes into the thermodynamically more stable α-anomer, and this anomerization is facilitated by Lewis acid additives.

**Scheme 17:**
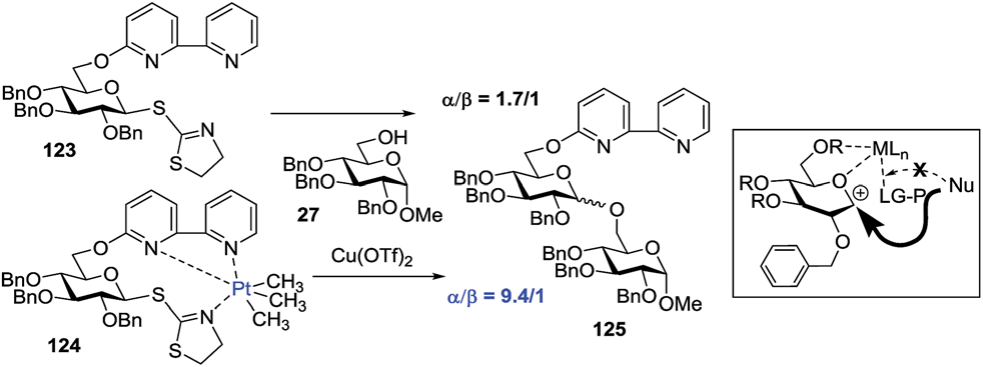
Effect of metal complexation on the stereoselectivity of glycosylation.

Demchenko and co-workers observed that multi-dentate metal coordination to the leaving group, along with a protecting group at *O*-6 and/or *O*-5, has a strong effect on the stereoselectivity of chemical glycosylation (Scheme 17). It was demonstrated that platinum(iv) complexation of 6-*O*-picolinyl or 6-*O*-bipyridyl to the leaving group, such as thiazolinyl, has a pronounced effect on the stereoselectivity of glycosylation.^119^ While the glycosidation of thioimidate donor **123** with acceptor **27** in the presence of Cu(OTf)_2_ gave disaccharide **125** with poor selectivity (α/β = 1.7/1), the complexed glycosyl donor counterpart **124** showed a significant 5-fold increase in 1,2-*cis* stereoselectivity (α/β = 9.4/1).

## F. Other effects and special methods

High pressure applied to reactions with participating glycosyl donors further enhances 1,2-*trans* selectivity;^120^ when the high pressure conditions were applied to glycosylation with a non-participating glycosyl donor, a remarkable increase in the reaction yield was noted with only marginal changes in stereoselectivity.^121^ Unfavorable steric interactions, such as “*double stereodifferentiation*”^122^ that occur between the glycosyl donor and acceptor in the transition state or other factors or conditions may unexpectedly govern the course and outcome of the glycosylation process.

A number of methods have been developed that do not include a formal glycosylation step.^123^ Typically, these indirect procedures include multistep syntheses and are of lower efficiency than direct glycosylation. Therefore, practical application of these techniques is envisaged for the synthesis of glycosidic linkages that cannot be easily accessed by conventional technologies. O'Doherty developed a well-rounded methodology for palladium(0)-catalyzed glycosylation, wherein carbohydrate chirality centers are installed post-glycosylationally.^25e,124^ The *de novo* asymmetric methodology was applied to the synthesis of mono, di, and oligosaccharides *via* a palladium-catalyzed reaction. The synthesis of 1,2-*cis* linkages have not yet been accomplished.

### F.1. Intramolecular aglycone delivery (IAD)

Barresi and Hindsgaul were the first to apply the idea of intramolecular glycosylation, which was used for the synthesis of β-mannosides.^125^ Subsequently, it was demonstrated that silicon bridge-mediated aglycone delivery provides high yields and excellent stereocontrol.^126^ Further improvements emerged with the introduction of the allyl-mediated strategy that affords high yields and complete stereoselectivity in α-glucosylation and β-mannosylation.^127^ More recently Ito and co-workers invented naphthylmethyl ether (NAP)-mediated intramolecular aglycone delivery that generally provides significantly higher yields in comparison to those of traditional approaches.^128^

**Scheme 18:**
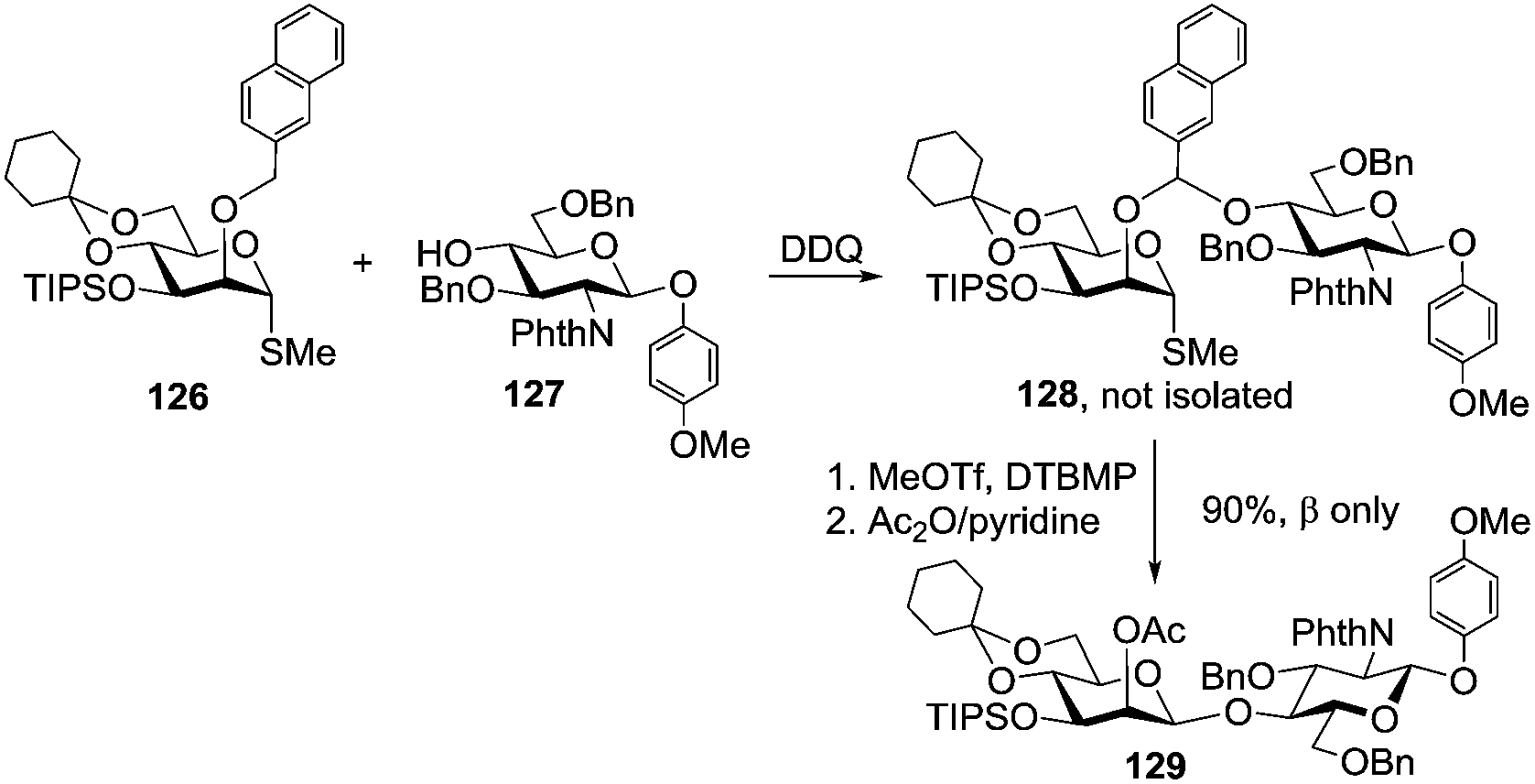
β-Mannosylation *via* NAP-tether mediated IAD.

A representative example, the synthesis of disaccharide **129**, is depicted in Scheme 18. Thus, when 2-*O*-NAP-protected thiomethyl glycosyl donor **126** was reacted with acceptor **127** in the presence of DDQ, followed by the removal of the NAP tether and acetylation, disaccharide **129** was obtained in 90% yield with complete β-selectivity. The further value of this methodology is that it allows for the stereoselective synthesis of various 1,2-*cis* linkages, such as β-Man*p*, β-Ara*f*, and α-Glc*p*.^129^

### F.2. Supported and tagged synthesis

**Scheme 19:**
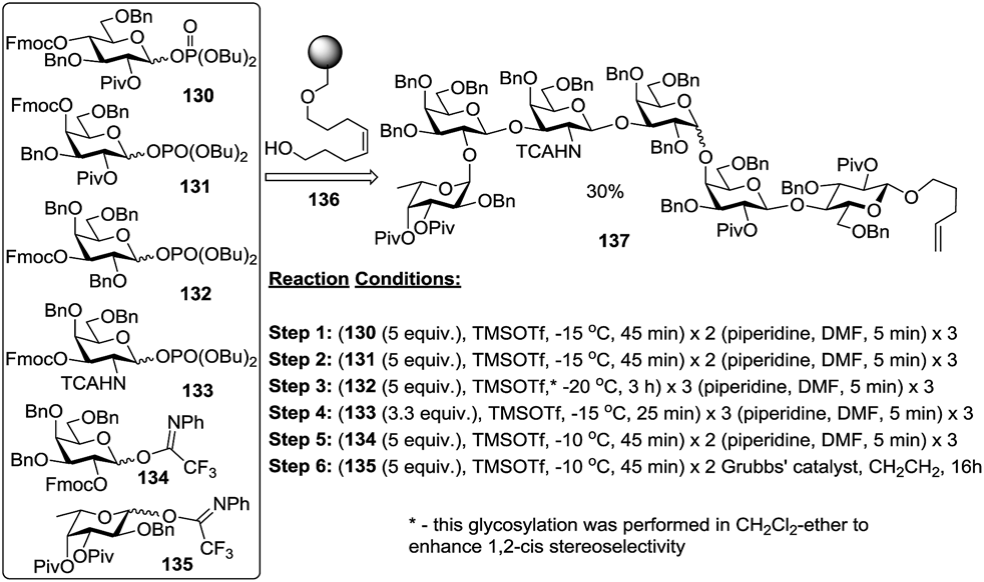
Automated synthesis of Globo H hexasaccharide.

The last decade has witnessed dramatic improvements in the area of solid phase-supported oligosaccharide synthesis.^130^ Polymer supported synthesis is very attractive because it allows execution of the synthesis of oligosaccharide sequences without the necessity of purifying (and characterizing) the intermediates. Another important advantage of oligosaccharide synthesis on solid phase supports is the ease of excess reagent removal (by filtration). This effort culminated in the automated synthesis by Seeberger, which was the first attempt to conquer the challenge of 1,2-*cis* glycosidic bond formation using an automated approach.^131^ Careful refinement of the reaction conditions allowed 1,2-*cis* galactosylation in dichloromethane-ether and a Globo-H sequence was assembled as depicted in Scheme 19. First, glycosyl phosphate donor **130** was linked to the resin **136** *via* glycosylation using TMSOTf (repeated once) as the promoter, followed by deprotection of the Fmoc substituent with piperidine (repeated twice) to provide a polymer-bound acceptor. The general synthetic protocol consists of repetitive cycles of glycosylation using either glycosyl phosphate (**130–133**) or PTFAI donors (**134** and **135**) followed by deprotection with piperidine. The final product **137** was obtained under an atmosphere of ethylene in the presence of Grubbs' catalyst^132^ in an overall yield of 30%.

Very recently the same group has reported the total synthesis of an *O*-antigen pentasaccharide repeating unit obtained from pathogenic *E. coli.* O111. With the synthetic challenge of constructing two unnatural and labile coitose units, the total synthesis was achieved in 21 steps with 1.5% overall yield.^133^ Boons *et al.* presented a very elegant synthesis of an α-linked oligosaccharide on a polymer support using their recent chiral auxiliary-assisted synthesis of 1,2-*cis* glycosides.^38^

**Scheme 20:**
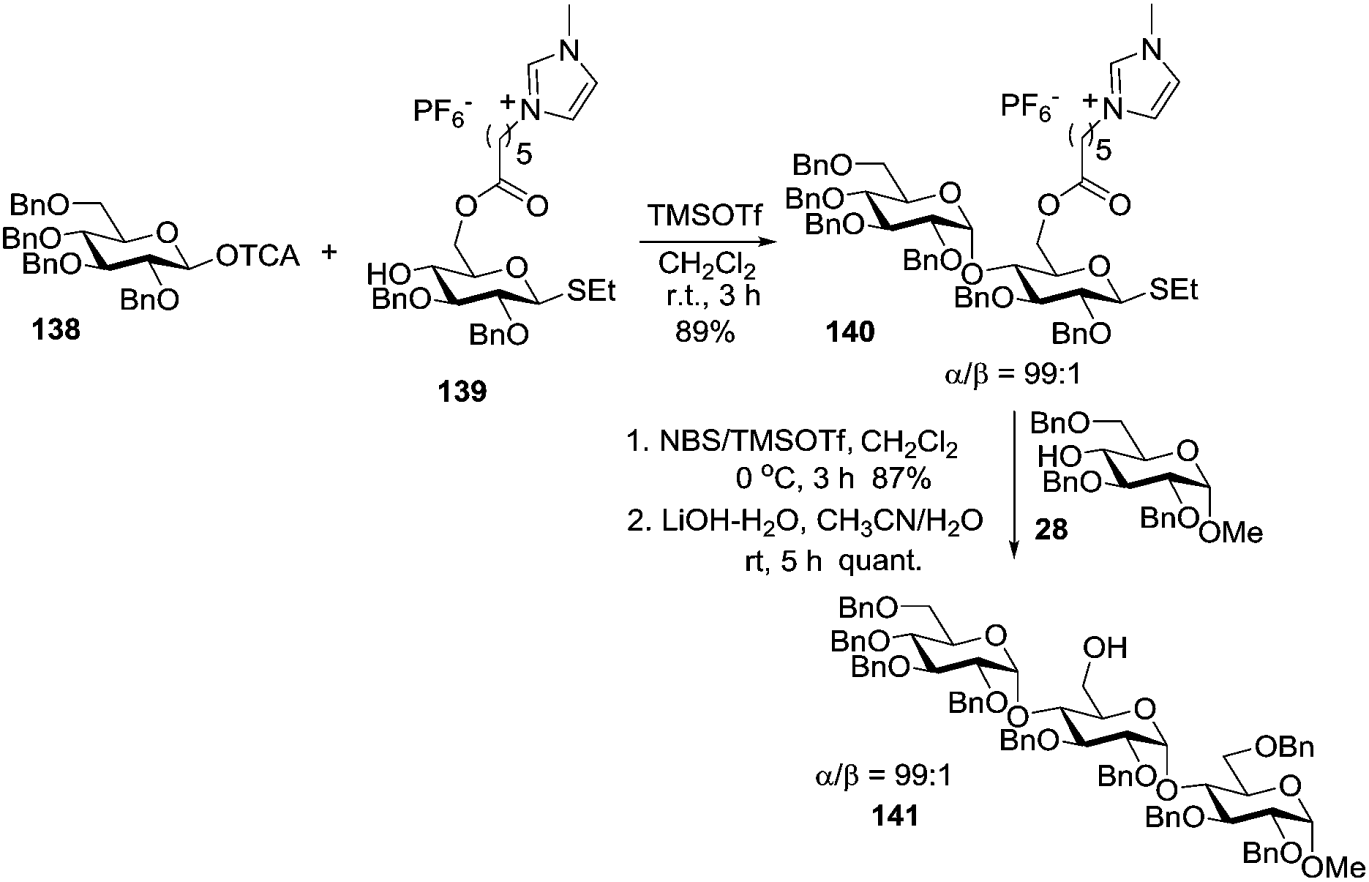
Glycosylation on an ionic liquid support.

A promising technique for tagged oligosaccharide synthesis that makes use of an ionic-liquid support has recently emerged.^134^ As with the polymer-supported and fluorous tag-supported syntheses,^135^ ionic liquid-supported assembly expedites oligosaccharide synthesis by eliminating the need for chromatographic purification of the intermediates.^134b,136^ Differently from insoluble polymer beads, ionic liquid supports allow for homogeneous conditions. This approach is illustrated by the synthesis of trisaccharide **141** (Scheme 20).^137^

In this synthetic strategy, the glycosyl acceptor **139** was grafted onto an ionic liquid support at the C-6 position of the sugar moiety. The resulting tagged glycosyl acceptor **139** was reacted with TCAI donor **138** to afford disaccharide **140** in 89% yield and high α-stereoselectivity. The purification is accomplished by simple washing or liquid–liquid extractions. Disaccharide **140** was then reacted with acceptor **28**, followed by the removal of the ionic liquid tag using LiOH–H_2_O to afford trisaccharide **141** in 87% yield.

## G. Conclusions and outlook

Progress in the area of chemical glycosylation has significantly improved our ability to synthesize various glycosidic linkages with impressive yields and stereoselectivity. Can we conclude that we have entirely solved the problem of chemical glycosylation? Unfortunately not, and hopefully this review has introduced the reader to the challenge of chemical glycosylation, a variety of factors, conditions, and driving forces influencing all aspects of this complex chemical reaction. Hopefully, the reader has obtained the information about specialized methods and strategies employed in modern carbohydrate chemistry.

The authors believe that progress in the development of new coupling methods and efficient strategies for oligosaccharide synthesis will ultimately provide an efficient and trouble-free access to complex saccharides. This goal cannot be achieved without comprehensive knowledge of the glycosylation mechanism and the driving forces of glycosylation and competing side processes. We project that subsequent scientific developments in this field will focus more and more on studying the mechanistic aspects of the glycosylation reaction. As new mechanistic knowledge emerges, further refinement of the reaction conditions and development of new directing protecting groups and even additional anomeric leaving groups may reemerge.
